# Upconversion nanoparticles@AgBiS_2_ core-shell nanoparticles with cancer-cell-specific cytotoxicity for combined photothermal and photodynamic therapy of cancers

**DOI:** 10.1016/j.bioactmat.2022.01.010

**Published:** 2022-01-10

**Authors:** Zhaoyou Chu, Tian Tian, Zhenchao Tao, Juan Yang, Benjin Chen, Hao Chen, Wanni Wang, Peiqun Yin, Xiaoping Xia, Hua Wang, Haisheng Qian

**Affiliations:** a School of Biomedical Engineering, School of Basic Medical Sciences, Anhui Provincial Institute of Translational Medicine, Anhui Medical University, Hefei, Anhui, 230032, PR China; b Department of Oncology, The First Affiliated Hospital of Anhui Medical University, Anhui Medical University, Hefei, Anhui, 230032, PR China; c Department of Radiation Oncology, The First Affiliated Hospital of USTC, Division of Life Sciences and Medicine, University of Science and Technology of China, Hefei, Anhui, 230031, China; d Department of Obstetrics and Gynecology, Children's Hospital of Anhui Medical University, Anhui Provincial Children's Hospital, Hefei, Anhui, 230051, PR China

**Keywords:** Upconversion nanoparticles, Core-shell nanoparticles, Photothermal conversion, Facile synthesis, Photodynamic therapy

## Abstract

UCNPs@AgBiS_2_ core-shell nanoparticles that AgBiS_2_ coated on the surface of upconversion nanoparticles (UCNPs) was successfully prepared through an ion exchange reaction. The photothermal conversion efficiency of AgBiS_2_ can be improved from 14.7% to 45% due to the cross relaxation between Nd ions and AgBiS_2_. The doping concentration of Nd ions played a critical role in the production of reactive oxygen species (ROS) and enhanced the photothermal conversion efficiency. The NaYF_4_:Yb/Er/Nd@NaYF_4_:Nd nanoparticles endows strong upconversion emissions when the doped concentration of Nd ions is 1% in the inner core, which excites the AgBiS_2_ shell to produce ROS for photodynamic therapy (PDT) of cancer cells. As a result, the as-prepared NaYF_4_:Yb/Er/Nd@NaYF_4_:Nd@AgBiS_2_ core-shell nanoparticles showed combined photothermal/photodynamic therapy (PTT/PDT) against malignant tumors. This work provides an alternative near-infrared light-active multimodal nanostructures for applications such as fighting against cancers.

## Highlights

• UCNPs@AgBiS_2_ core-shell nanoparticles firstly prepared via a facile chemical process.
• The photothermal conversion efficiency for UCNPs@AgBiS_2_ core-shell nanoparticles reached up to 45%.
• The as-prepared UCNPs@AgBiS_2_ core-shell nanoparticles showed superoroir therapeutic efficacy for cancers.

## Graphical abstract

Uniform upconversion nanoparticles (UCNPs)@AgBiS_2_ core-shell nanoparticles were successfully prepared, which showed enhanced photothermal conversion performance and good reactive oxygen species (ROS) production ability for combined photothermal-photodynamic (PTT-PDT) therapy of cancer.

**Figure:**
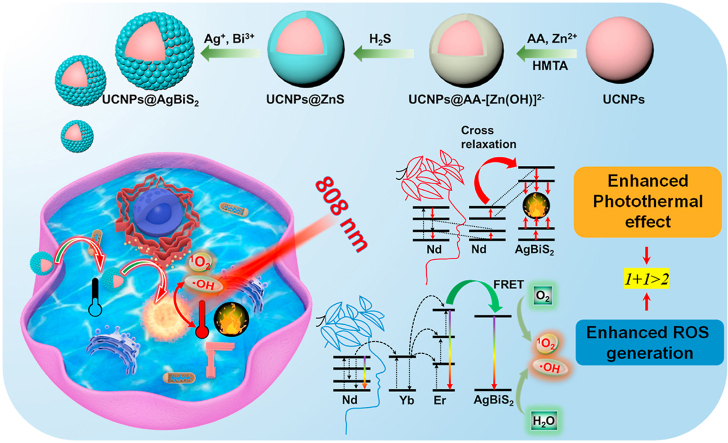

## 1 Introduction

During the past two decades, lanthanide ion-doped upconversion nanoparticles (UCNPs) have attracted tremendous attention owing to their unique capability to generate shorter wavelength emissions under the excitation of longer wavelengths [[1], [2], [3], [4], [5]]. Especially, UCNPs have been recognized as one kind of energy transducer for producing reactive oxygen species (ROS), enhancing energy migration for various applications, such as photodynamic therapy (PDT) [6,7], photothermal therapy (PTT) [8,9], and controlled drug delivery [[10], [11], [12]]. Among these, PDT has aroused great research interest in recent years due to its low systemic toxicity and minimal invasiveness. However, PDT for cancer treatment is hampered by tumor hypoxia, which involves sufficient oxygen, photosensitization and light excitation [13,14]. In addition, the traditional organic photosensitizer is inefficient to produce active oxygen and poor chemical stability, which leads to low efficacy for PDT in cancer treatment. After enormous efforts have been devoted, it was found that the combination of UCNPs with semiconductors can achieve the ideal efficacy of PDT to overcome oxygen dependence [[15], [16], [17]].

Additionally, a variety of semiconductors with excellent photothermal conversion abilities, including CuS, Bi_2_S_3_, gold (Au), carbon and metal chalcogenides, have been combined with UCNPs to achieve excellent photothermal and photodynamic efficacy [[18], [19], [20], [21], [22], [23], [24]]. In particular, bismuth (Bi)-based nanomaterials, such as Bi, Bi_2_S_3_, AgBiS_2_ and Bi_2_Se_3,_ have been proven to be promising candidates as superior photothermal conversion agents, owing to their light absorption coefficient, heat dissipation rate and photothermal conversion efficiency [[25], [26], [27], [28], [29], [30]]. Furthermore, Bi-based nanomaterials with a narrow band gap can also be used as catalytic materials, which have the ability to generate ROS under light irradiation. In the past few years, photothermal-enhanced photodynamic therapy has been recognized as efficient and non-invasive modalities for cancer treatment since thermal effects at an appropriate level can increase intratumoral blood flow and subsequently transport more oxygen into the tumor, resulting into yielding synergistic or combined therapeutic outcomes even in severely hypoxic solid tumors [31,32].

**Scheme 1:**
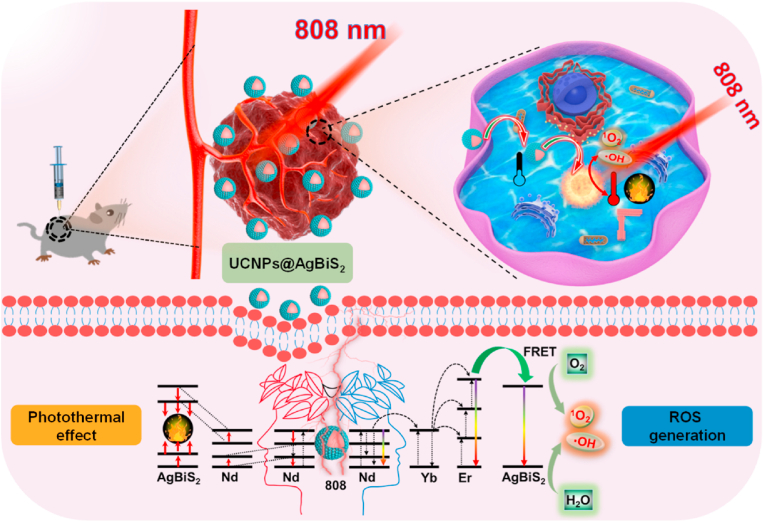
UCNPs@AgBiS_2_ core-shell nanoparticles were constructed for NIR-activated combined PTT-PDT of cancer. The photothermal conversion ability and ROS production capability could be tuned based on two different modes (up-/down-conversion luminescence).

AgBiS_2_ hollow nanospheres exhibited excellent chemical stability and good cancer-cell-specific cytotoxicity, which was synthesized by our previous reported protocol [33]. Herein, we proposed an efficient strategy to fabricate AgBiS_2_-coated Nd^3+^-sensitized upconversion nanoparticles (denoted UCNPs) to construct unique UCNPs@AgBiS_2_ core-shell nanoparticles (NPs) for enhanced photothermal conversion efficiency owing to the potential cross-relaxation pathways between the continuous energy band of AgBiS_2_ and the ladder-like energy levels of Nd^3+^ ions (Scheme 1). Steady/transient state fluorescence spectroscopy has been employed to demonstrate the energy migration mechanism and the cross-relaxation pathways [34]. The ROS production capability and photothermal conversion ability have been studied based on two different modes (up-/down-conversion luminescence). Antitumor experiments *in vitro* and *in vivo* were conducted upon 808 nm laser irradiation to demonstrate the privilege of the core-shell NPs. As expected, the as-prepared UCNPs@AgBiS_2_ core-shell nanoparticles (NPs) with cancer-cell-specific cytotoxicity would show suporior therapeutic efficacy.

## 2 Materials and methods

### 2.1 Materials

NaNdF_4_@NaYF_4_:Nd_0.2_ (abbreviated as Nd@Nd), NaYF_4_:Yb_0.3_/Er_0.005_/Nd_x_ (X = 0, 0.005, 0.01, 0.03, 0.05)@NaYF_4_:Nd_0.2_ (abbreviated as UCNPs) core-shell NPs, UCNPs@AA-[Zn(OH)_4_]^2-^ and UCNPs@ZnS nanoparticles showed good dispersability and were prepared according to our protocol reported previously [35]. Other chemicals were of analytic grade and used as received.

### 2.2 Synthesis of UCNPs@AgBiS_2_ core-shell NPs

In a typical procedure, 0.045 mmol UCNPs@ZnS was added to 10 mL of ethylene glycol solution containing 0.09 mmol of thiourea to form milky dispersions. Subsequently, the solution was slowly heated to 130 °C under stirring. Then, AgNO_3_ and Bi(NO_3_)_3_ (0.045 mmol) ethylene glycol solution were added to the above solution under constant stirring, which was maintained at 130 °C for 10 min. UCNPs@AgBiS_2_ core-shell NPs were obtained by washing three times with ethanol and deionized water.

### 2.3 Cytotoxicity experiment

Typically, 4T1 (mouse breast cancer cells) cells were seeded in a 96-well plate at 1 × 10^4^ cells/well and then incubated with different concentrations of UCNPs@AgBiS_2_ (0, 10, 20, 40, 80, and 160 μg mL^−1^) for 12 h. Subsequently, laser irradiation (808 nm, 1 W cm^−2^) was performed for different times (0, 1, and 3 min), addition of 3-[4,5-dimethylthiazol-2-yl]-2,5-diphenyltetrazolium bromide (MTT) solution and incubation for 4 h to form formazan. Finally, 100 μL dimethyl sulfoxide was added to dissolve and measure the absorbance at 570 nm with a microplate reader to determine the relative cell viability.

### 2.4 In vitro ROS assay

Typically, 4T1 cells were seeded in a 6-well plate at 1 × 10^5^ cells/well and then incubated with UCNPs@AgBiS_2_ (50 μg mL^−1^) for 12 h. Subsequently, the cells were irradiated with an 808 nm laser (1 W cm^−2^) for 3 min. Then, DCFH-DA (2′,7′-dichlorodihydrofluorescein diacetate) was incubated for 0.5 h to form green fluorescent substance (DCF). Finally, the intracellular green fluorescence was monitored by confocal laser scanning microscopy (CLSM) and flow cytometry analysis.

### 2.5 Live/dead cell staning

Typically, 4T1 cells were seeded in a 24-well plate at 5 × 10^4^ cells/well and then incubated with UCNPs@AgBiS_2_ (50 μg mL^−1^) for 12 h. Subsequently, the cells were irradiated with an 808 nm laser (1 W cm^−2^) for 3 min. Then, calcein (AM) and propidium iodide (PI) were added for the staining of living and dead 4T1 cells and incubated to form different fluorescent substances. Digital fluorescence photographs of the cells were captured using a fluorescence microscope.

### 2.6 Apoptosis

Apoptosis quantitatively explored by flow cytometry. Usually, 4T1 cells were seeded into a 6-well plate at 1 × 10^5^ cells/well and then incubated with UCNPs@AgBiS_2_ (50 μg mL^−1^) for 12 h. Subsequently, the cells were irradiated with an 808 nm laser (1 W cm^−2^) for 3 min. After that, the cells were digested with trypsin and stained with Annexin V-FITC/PI, and the rate of apoptosis was quantitatively determined by flow cytometry analysis.

### 2.7 Characterization

The surface morphology, phase, fluorescence, optical properties, X-ray diffraction (XRD) and X-ray photoelectron spectra (XPS) of these products were investigated carefully according to our previous protocol or instruments [33]. Photothermal performance, ROS, hydroxyl radical (·OH) and singlet oxygen (^1^O_2_) detection were studied via our previously reported protocol. All animal experimental protocols were investigated carefully according to our previous protocol or instruments. All animal experiments were approved by the Ethical Committee of Anhui Medical University (approved number: LLSC20210077).

## 3 Results and discussion

### 3.1 Synthesis and characterization of UCNPs@AgBiS_2_ core-shell NPs

**Fig. 1:**
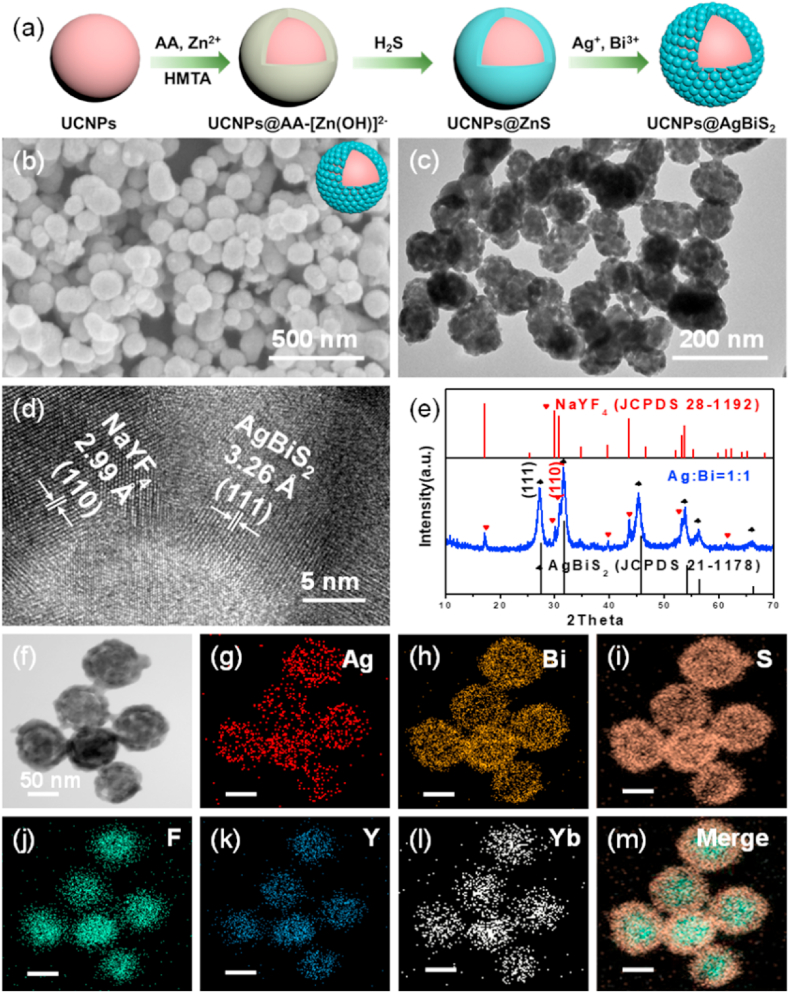
(a) Schematic illustration of the synthesis of UCNPs@AgBiS_2_ core-shell NPs. (b) FESEM, (c) TEM and (d) HRTEM images, (e) XRD pattern of UCNPs@AgBiS_2_ core-shell NPs. (f) Representative STEM image and (g–m) elemental mapping images of Ag, Bi, S, F, Y, Yb and merged image of UCNPs@AgBiS_2_ core-shell NPs.

UCNPs@AgBiS_2_ NPs were fabricated via an ion exchange reaction using UCNPs@ZnS core-shell NPs as sacrificed templates **(**Fig. 1a). UCNPs and UCNPs@AA-[Zn(OH)_4_]^2-^ and UCNPs@ZnS NPs with uniform morphology and excellent dispersion were synthesized according to our previous report (Fig. S1, supporting information) [35]. Fig. 1b and c showed that the as-prepared UCNPs@AgBiS_2_ NPs consisted of uniform spherical structure with an average size in approximately 80 nm. In the high-resolution transmission electron microscopy (HRTEM) image taken from the marginal area of UCNPs@AgBiS_2_ NPs, lattice spacings of 2.99 and 3.26 Å were assigned to the (110) and (111) planes of hexagonal NaYF_4_ and cubic AgBiS_2_, respectively (Fig. 1d) [33]. Moreover, XRD patterns of the final samples were shown in Fig. 1e, **which confirmed that the sample was composed of the cubic phase of AgBiS**_**2**_ **(JCPDS No. 21**–**1178) and hexagonal NaYF**_**4**_ **(JCPDS No. 28**–**1192). Therefore, based on the above analysis, AgBiS**_**2**_ **was proven to be successfully coated on the surface of UCNPs. Furthermore, as displayed in** Fig. 1f–m and Fig. S2, the corresponding elements were confirmed by elemental mapping, and Ag, Bi, and S elemental signals were captured in the outer shell, while the other elements were detected inside, indicating that core-shell NPs were apparent with hexagonal UCNPs inside (∼50 nm diameter) and the shell layer of AgBiS_2_ (∼30 nm shell thickness). The dynamic light scattering (DLS) size of UCNPs@AgBiS_2_ was around 100 nm and the zeta potential was −3.45 ± 0.4 mW (Fig. S3). The content of UCNPs@AgBiS_2_-related elements (e.g., F, Na, S, Y, Ag, Nd, Yb, Er, and Bi) was detected by Energy Dispersive X-Ray Spectroscopy (EDX) analysis (Table S1). The surface components and chemical states of the elements were further elucidated by XPS, in which the survey spectrum indicated that the as-prepared materials contained Ag, Bi, S, Y, Na, F, Yb, Nd and Er elements (Fig. S4). In addition, the binding energies of Ag located at 367.90 and 374.27 eV were deconvoluted to 3d_5/2_ and 3d_3/2_. Meanwhile, the peaks at binding energies of 158.38 and 163.68 eV can be attributed to Bi 4f_7/2_ and 4f_5/2_, respectively, indicating the successful formation of pure AgBiS_2_ NPs. Moreover, the binding energies for Y, Na, F, Yb, Nd and Er were weak, which was also clearly observed because of UCNPs embedded in the shell layer of AgBiS_2_. These results coherently verified that UCNPs@AgBiS_2_ core-shell NPs were successfully fabricated.

### 3.2 Optical and photothermal properties of UCNPs@AgBiS_2_ core-shell NPs

**Fig. 2:**
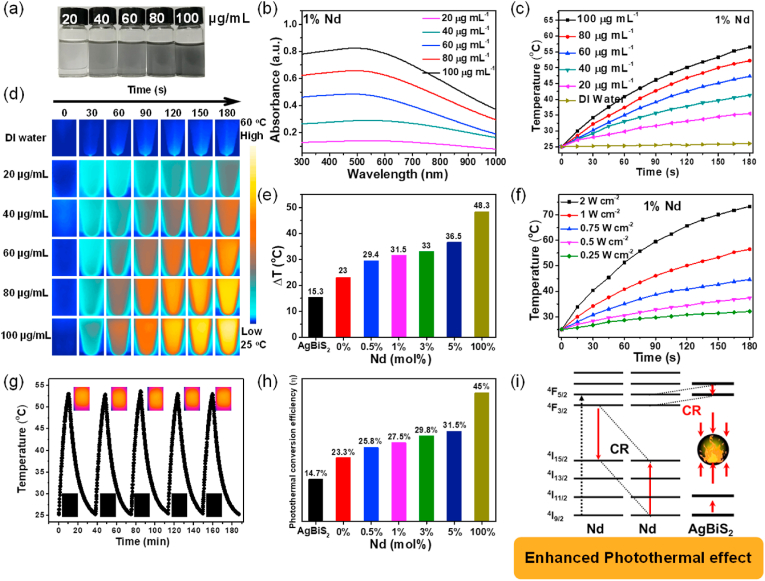
(a) Digital photograph of UCNPs@AgBiS_2_ at various concentrations. (b) UV–Vis–NIR absorption spectra of UCNPs@AgBiS_2_ dispersions at various concentrations. (c) Photothermal heating curve of UCNPs@AgBiS_2_ dispersions at various concentrations. (d) Thermal images of UCNPs@AgBiS_2_ solutions at various concentrations upon 808 nm laser irradiation (1 W cm^−2^). (e) Temperature change of different nanoparticles with different Nd doping concentrations under 808 nm laser irradiation. (f) Temperature changes of UCNPs@AgBiS_2_ dispersions under 808 nm laser irradiation at various power densities. (g) Heating/cooling curve of UCNPs@AgBiS_2_ nanoparticles after repeatedly turning on/off laser irradiation for five cycles. (h) Photothermal conversion efficiency (η) change of different nanoparticles with different Nd doping concentrations under laser on/off. (i) Schematic illustration of the generation of cross-relaxation pathways between Nd^3+^ ions and AgBiS_2_.

Desired NIR absorption and excellent photothermal conversion efficiency are the basis for the use of photothermal reagents in PTT. Fig. 2a showed digital photographs of UCNPs@AgBiS_2_ aqueous solutions with different concentrations, and the color deepened as the concentration increased. According to the corresponding UV–vis–NIR absorption spectrum (Fig. 2b), UCNPs@AgBiS_2_ NPs with different concentrations showed wide and strong optical absorption in the NIR region, which proved the possibility of their high photothermal conversion performance. The distinguished photothermal effects with various concentrations of UCNPs@AgBiS_2_ NPs were investigated by an infrared camera with an 808 nm laser. Upon 808 nm laser irradiation, the temperature of the UCNPs@AgBiS_2_ NPs increased rapidly over time, indicating that UCNPs@AgBiS_2_ NPs had excellent photothermal effects (Fig. 2c–e, and Fig. S5). When the concentration of UCNPs@AgBiS_2_ suspension solution was kept at 100 μg mL^−1^, the temperature increased from 25 to 56.5 °C after irradiation for 3 min. In contrast, only a faint increase of 2.3 °C took place for deionized water as a control group. Moreover, the power controllability and photothermal stability of UCNPs@AgBiS_2_ NPs were explored, and the temperature of the UCNPs@AgBiS_2_ aqueous solution increased with laser power (Fig. 2f and g). Then, five laser ON/OFF cycles were employed to investigate the photostability of UCNPs@AgBiS_2_ core-shell NPs. In Fig. 2g and Fig. S6, after five laser cycles, the photothermal effect of UCNPs@AgBiS_2_ showed almost no obvious attenuation, highlighting its outstanding photostability. The time constant was 417.86 s, as shown in Fig. S7, the photothermal conversion efficiency (*η*) of the UCNPs@AgBiS_2_ (1% Nd) aqueous solution was calculated to be 27.5% on the basis of the heating-cooling profile, and the photothermal conversion efficiency increased with the Nd concentration (Fig. 2h). In contrast, the temperature of the Nd@Nd@AgBiS_2_ (100% Nd) suspension with a concentration of 100 μg mL^−1^ was increased from 25 to 80.2 °C. As shown in Fig. 2h and Fig. S8, the photothermal conversion efficiency of Nd@Nd@AgBiS_2_ core-shell NPs were calculated to be 45.0%, which was higher than that of most widely studied PTT agents, such as Bi_2_S_3_ (26.8%, 28.1%), AgBiS_2_-TPP (23.5%), AgBiS_2_-PEI (21.3%), AgBiS_2_-PEI (35.2%) and AgBiS_2_ (36.51%) (Table S2) [[26], [27], [28], [29],^36^,^37^]. The doping of Nd ions facilitated the energy transfer and conversion of the as-prepared core-shell NPs, which could be attributed to the cross-relaxation (CR) process between Nd^3+^ ions and AgBiS_2_ (Fig. S9). The photons could be excited to the ^4^F_5/2_ state and then descend to the ^4^F_3/2_ state via non-radiation when the Nd^3+^ ions were exposed to 808 nm irradiation (Fig. 2i) [38,39]. In the ^4^F_3/2_ state, the photon can be attenuated to a lower energy state by the irradiation process, resulting in 900 nm (^4^F_3/2_ to ^4^I_9/2_), 1058 nm (^4^F_3/2_ to ^4^I_11/2_), and 1332 nm (^4^F_3/2_ to ^4^I_13/2_) emission. CR between the ^4^F_3/2_ to ^4^I_15/2_ and ^4^I_9/2_ to ^4^I_15/2_ states of different Nd^3+^ ions and other non-irradiative transitioned to the ground state produces photothermal effects. When the photons in Nd@Nd dropped from ^4^F_5/2_ to ^4^F_3/2_, the photons in AgBiS_2_ could jump from the lower level to the higher level with the same energy difference as between ^4^F_5/2_ and ^4^F_3/2_, thus forming a shorter CR2 path, which can generate more heat energy in Nd@Nd. Moreover, the photothermal performance of UCNPs@AgBiS_2_ core-shell NPs could be adjusted by changing the concentration of Nd ions. Taken together, these results demonstrated that UCNPs@AgBiS_2_ NPs are excellent photothermal agents with outstanding photothermal performance under NIR laser irradiation.

### 3.3 ROS production and detection for the UCNPs@AgBiS_2_ core-shell NPs

**Fig. 3:**
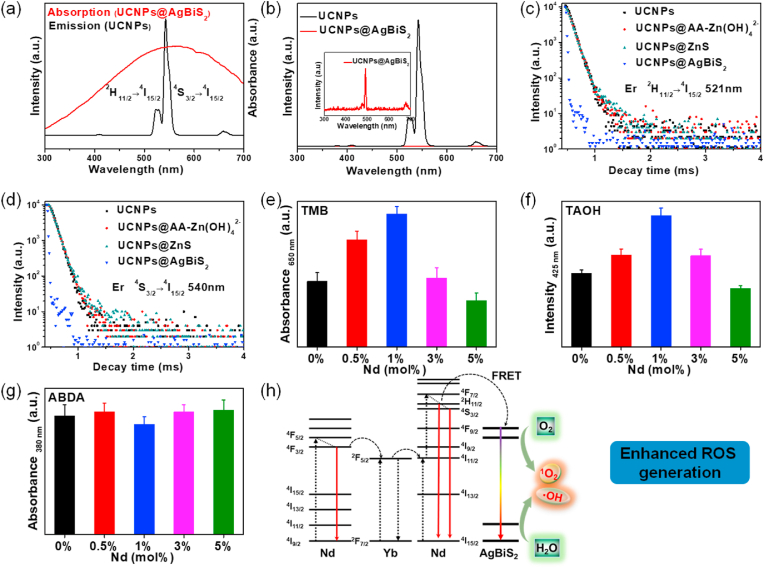
(a) The overlapping spectrum between the UV–Vis absorption spectra of UCNPs@AgBiS_2_ core-shell nanoparticles and the fluorescence spectra of UCNPs. (b) Fluorescence spectra of the UCNPs and UCNPs@AgBiS_2_ core-shell nanoparticles. The inset shows the corresponding enlarged images of UCNPs@AgBiS_2_. (c, d) The luminescence decays of the excited state levels of Er^3+^ at 521 and 540 nm for the UCNPs, UCNPs@AA-[Zn(OH)_4_]^2-^, UCNPs@ZnS, and UCNPs@AgBiS_2_ core-shell nanoparticles, respectively. (e) Absorbance change at 650 nm of TMB in the presence of different nanoparticles with different Nd doping concentrations under 808 nm laser irradiation. (f) Intensity change at 425 nm of TAOH in the presence of different nanoparticles with different Nd doping concentrations under 808 nm laser irradiation, indicating the production of ·OH species. (g) Absorbance change at 380 nm of ABDA in the presence of different nanoparticles with different Nd doping concentrations under 808 nm laser irradiation, indicating the production of ^1^O_2_ species. (h) Schematic illustration of the enhanced generation of ROS between Er^3+^ ions and AgBiS_2_.

The unique narrow band gap not only gives AgBiS_2_ photothermal properties but also endows it the potential for ROS generation. As shown in Fig. 3a and Fig. S10, UCNPs@AgBiS_2_ core-shell NPs possessed a wider and larger absorption peak at 550 nm, which indicated that the absorption peak of AgBiS_2_ matched the upconversion luminescence (UCL) emission of the ^4^S_3/2_ → ^4^I_15/2_ and ^2^H_11/2_ → ^4^I_15/2_ transitions of Er^3+^ [40]. The fluorescence emissions for the UCNPs@AgBiS_2_ core-shell NPs were completely quenched (Fig. 3b), demonstrating the enhancement of fluorescence resonance energy transfer (FRET) efficiency between the UCNPs core and the AgBiS_2_ shell, which agreed well with the aforementioned results. To further investigate the FRET efficiency of UCNPs@AgBiS_2_ core-shell NPs upon 808 nm NIR laser excitation, the luminescence decays of the excited state levels of Er^3+^ were detected for the UCNPs, UCNPs@AA-[Zn(OH)_4_]^2-^, UCNPs@ZnS and UCNPs@AgBiS_2_ NPs at 521 and 540 nm, respectively (Fig. 3c and d). Compared with UCNPs, UCNPs@AA-[Zn(OH)_4_]^2-^ and UCNPs@ZnS, the FRET effect between UCNPs and AgBiS_2_ was greatly enhanced, which demonstrated that high FRET efficiency was achieved. Previous studies revealed that composite nanostructure-incorporated UCNPs and semiconductors showed desired ROS production ability under NIR laser irradiation owing to their energy transfer between the core and shell components [41]. Then, 3,5,3,5-tetramethylbenzidine (TMB) was used to detect the generation of ROS, and the absorbance increased after the addition of UCNPs@AgBiS_2_ NPs, indicative of ROS generation (Fig. 3e and Fig. S11). Furthermore, the ROS species were confirmed by terephthalic acid (TAOH), and 9,10-anthracenediyl-bis(methylene) dimalonic acid (ABDA). ·OH was confirmed by fluorescence spectra under the probe of TAOH, and ^1^O_2_ was detected by UV–Vis spectra to verify the destruction of ABDA [33,42]. As shown in Fig. 3f-g and Fig. S12-13, the fluorescence intensity at 425 nm of TAOH increased and the absorption intensity at 380 nm of ABDA decreased with illumination time, indicating that UCNPs@AgBiS_2_ could produce large amounts of ROS under 808 nm laser irradiation. Although AgBiS_2_ theoretically had the ability to generate ROS under 808 nm laser irradiation, the ROS generated by pure AgBiS_2_ was far less than that of UCNPs@AgBiS_2_ under the same conditions [37]. In general, UCNPs@AgBiS_2_ NPs excited by an 808 nm laser displayed a favorable ability to produce ROS for PDT, which may be ascribed to the excellent energy transfer between AgBiS_2_ and UCNPs. Based on the above results, the proposed ROS generation mechanism under the NIR response was proposed, including continuous Nd^3+^ → Yb^3+^ → activator energy transfer, which activated AgBiS_2_ through energy transfer to generate ROS (Fig. 3h). First, Nd^3+^ ions in the active core/shell UCNPs were excited to the ^4^F_5/2_ state under 808 nm laser irradiation and then relaxed to the ^4^F_3/2_ state under non-irradiation conditions. Energy could be transferred through the shell to nearby Yb^3+^ ions and filled into their ^2^F_5/2_ state, which eventually acted as an effective bridge to relay the energy to Er^3+^ ions. Additionally, this energy transfer path initiated a typical upconversion process in the core, where Er^3+^ ions were excited to high energy levels, such as ^4^F_9/2_, ^4^S_3/2_, and ^2^H_11/2_. The excited electron of Er^3+^ relaxed into the ground state and emitted green light [[43], [44], [45]]. AgBiS_2_ could be activated by means of FRET and then react with O_2_ and H_2_O in the surrounding environment to produce ·OH and ^1^O_2_. As expected, the as-prepared UCNPs@AgBiS_2_ core-shell NPs possessed excellent ROS generation performance for ^1^O_2_ and ·OH to kill tumor cells by combining PTT and PDT.

### 3.4 *In vitro* NIR activated PTT-PDT

**Fig. 4:**
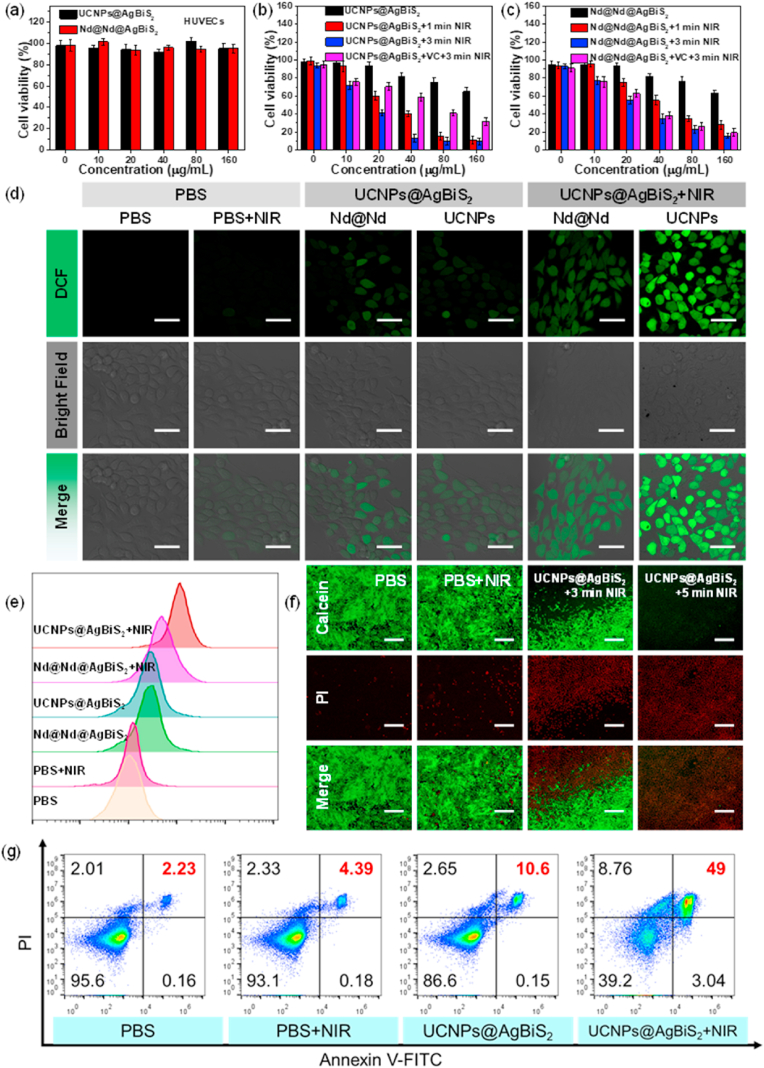
(a) Cell toxicity of HUVECs after incubation with various concentrations of UCNPs@AgBiS_2_ and Nd@Nd@AgBiS_2_. (b–c) Apoptosis ablation of 4T1 cells incubated with various concentrations of UCNPs@AgBiS_2_ and Nd@Nd@AgBiS_2_ under irradiation (808 nm, 1.0 W cm^−2^). ROS analysis of 4T1 cells stained by DCFH-DA. (d) CLSM images and (e) flow cytometry analysis. Scale bar = 50 μm. (f) Fluorescence microscopy images of 4T1 cells after different treatments as indicated. Scale bar = 500 μm. (g) Flow cytometric analysis of 4T1 cell apoptosis induced by different treatments with Annexin V-FITC/PI staining.

The excellent photothermal/photodynamic effects of UCNPs@AgBiS_2_ core-shell NPs prompted us to study their killing effect on cancer cells. Fig. S14 showed the confocal laser scanning microscopy (CLSM) images of the cocultivation of Nile Red (NR) loaded UCNPs@AgBiS_2_ with 4T1 cells, in which the concentration of UCNPs@AgBiS_2_-NR were increased, the red fluorescence of NR continued to deepen in the cells, proving UCNPs@AgBiS_2_ has cells ability to internalize. Then, human umbilical vein endothelial cells (HUVECs) and mouse mammary epithelium cells (HC11) were employed as a normal cell model to evaluate the cytotoxic effect of UCNPs@AgBiS_2_ and Nd@Nd@AgBiS_2_ by MTT assay [46]. As shown in Fig. 4a and Fig. S15, cell viability was maintained above 95%, while the concentration of UCNPs@AgBiS_2_ was up to 160 μg mL^−1^. In addition, the spectrophotometric method with 3,5-Br_2_-PADAP was adopted to evaluate the leakage of Ag ions from UCNPs@AgBiS_2_ within 24 h in aqueous solution [[46], [47]]. It was revealed that the UCNPs@AgBiS_2_ showed superior chemical stability, and no leakage of Ag ions was observed (Fig. S16). *In vitro* cytotoxic effects of UCNPs@AgBiS_2_ on 4T1 cells were implemented under NIR at different times (0, 1, and 3 min). The cell viability decreased slightly when treated with only UCNPs@AgBiS_2_ or Nd@Nd@AgBiS_2_, which could be attributed to the cell-specific cytotoxicity of AgBiS_2_ (Fig. 4b-c and Fig. S17) [33]. Furthermore, almost no cells remained alive when the concentration was up to 160 μg mL^−1^ under irradiation for 3 min. In contrast, the cells still maintained a high survival rate without NIR laser irradiation. In particular, the UCNPs@AgBiS_2_ core-shell NPs showed the best therapeutic efficacy toward cancer cells owing to the production of ROS compared to Nd@Nd@AgBiS_2_ core-shell NPs. Ascorbic acid (VC, 100 μg mL^−1^), as a reducing agent, has been used to protect the cells against ROS and was added to 4T1 cells treated with UCNPs@AgBiS_2_ upon irradiation with an NIR laser [48]. The Nd@Nd@AgBiS_2_ core-shell NPs demonstrated better photothermal therapeutic efficacy, which could be attributed to their superior photothermal effect. Herein, UCNPs@AgBiS_2_ core-shell NPs showed extraordinary therapeutic efficiency for combined PTT-PDT of cancer cells [[49], [50], [51]]. The CLSM images forthrightly demonstrated the production of ROS within the cell, which was due to the green fluorescence of DCF converted from the nonfluorescent ROS probe DCFH-DA (Fig. 4d) [52]. No green fluorescence representing ROS was observed when only treatment with PBS or NIR laser. When UCNPs@AgBiS_2_ or Nd@Nd@AgBiS_2_ was added, slight green fluorescence in 4T1 cells was observed, indicating that UCNPs@AgBiS_2_ had the ability to catalyze H_2_O_2_ in the tumor microenvironment to generate ROS, which corresponded with the results of the cell experiments. The CLSM images illustrated the highest green fluorescence in the cytoplasm of the irradiated group. In addition, the UCNPs@AgBiS_2_ group showed stronger green fluorescence than the Nd@Nd@AgBiS_2_ group, suggesting that UCNPs could convert 808 nm light into higher energy visible light to excite AgBiS_2_ with a low bandgap to produce more ROS. In addition, the flow cytometry techniques were also used to quantitatively analyze the production of intracellular ROS. In the presence of UCNPs@AgBiS_2_ or Nd@Nd@AgBiS_2_, the fluorescence intensity of DCF increased slightly, and the fluorescence intensity of the irradiated group was higher than that of the nonirradiated group, which was consistent with the CLSM images (Fig. 4e). A large amount of reinforced lethal intracellular ROS was generated by laser excitation of UCNPs@AgBiS_2_, which was concluded by the results of CLSM observation and flow cytometry analysis [53]. Furthermore, the cancer cell killing effect of UCNPs@AgBiS_2_ was investigated by living and dead cell staining with Calcein-AM and propidium iodide (PI) [54]. Fluorescence imaging of 4T1 cells costained with calcein-AM and propidium iodide (PI) revealed that UCNPs@AgBiS_2_ core-shell NPs caused cancer cell death in a laser time-dependent manner (Fig. 4f). Finally, the flow cytometry with Annexin V-fluorescein isothiocyanate/propidium iodide (FITC/PI) staining was used to analyze apoptosis. As shown in Fig. 4g, the results showed that apoptosis was greatly increased in the presence of both NIR and UCNPs@AgBiS_2_. The above data fully demonstrated that UCNPs@AgBiS_2_ could efficiently kill cancer cells by combining PTT and PDT. Collectively, these results indicated that UCNPs@AgBiS_2_ could efficiently convert 808 nm light into higher energy visible light to excite AgBiS_2_, then increasing the production of ROS, thereby achieving a higher cell killing effect.

### 3.5 *In vivo* combined PTT-PDT

**Fig. 5:**
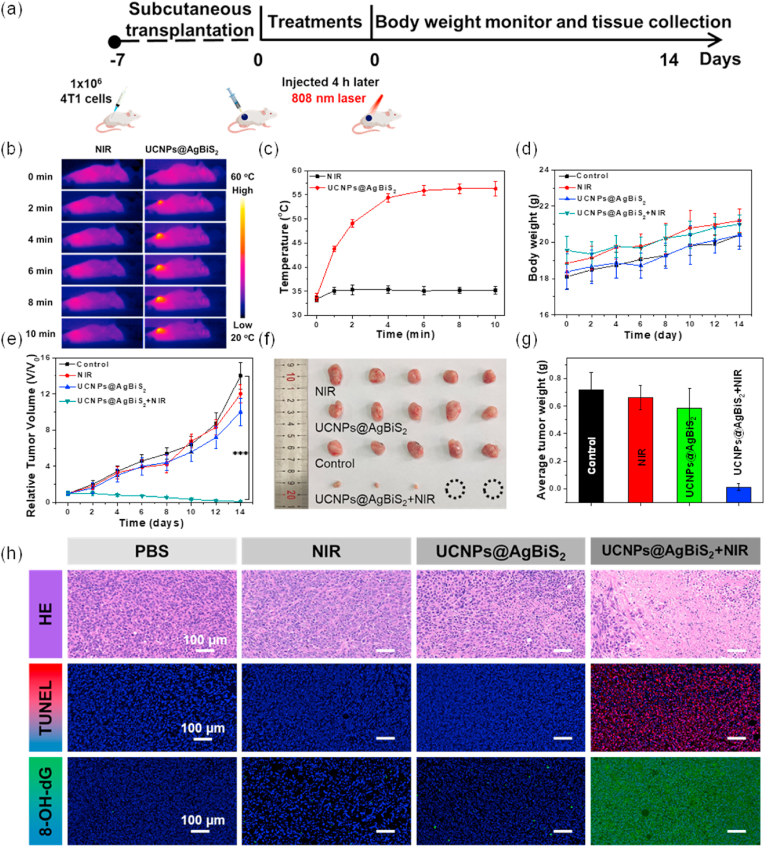
(a) Schematic illustration of tumor therapeutic profile. (b) IR thermal images in the control groups and UCNPs@AgBiS_2_-treated group at different time intervals after intravenous injection and (c) corresponding temperature curves. (d) Body weight profile. (e) Relative tumor growth curves. (f) Corresponding excised tumor photographs. (g) Mean excised tumor weights. (h) H&E, TUNEL and 8-OH-dG staining images of excised tumors of different treatment groups on the 14th day. (Statistical analysis was performed using a *t*-test: ***P < 0.001).

Encouraged by the excellent effect of UCNPs@AgBiS_2_ NPs against 4T1 cells, 4T1 tumor-bearing BALB/c mice were employed to investigate the *in vivo* phototherapeutic effect of USP. The *in vivo* thermal behaviors of UCNPs@AgBiS_2_ were accurately evaluated by tracking the heat signal under an infrared thermal camera on 4T1 tumor mice (Fig. 5a-c) [55]. The *in-situ* temperature of the tumors treated with UCNPs@AgBiS_2_ rapidly increased under 808 nm laser irradiation (0.5 W cm^−2^, 10 min). Because the temperature of the tumor regions reached 56.3 °C, the cells in tumor sites were ablated, and their malignant proliferation was inhibited effectively. In contrast, the temperature of tumors in the control group was not obviously changed. Inspired by the excellent photothermal effect of UCNPs@AgBiS_2_ *in vivo*, the *in vivo* tumor growth inhibition effect was investigated after intratumoral injection. When the volume of the tumor reached around 100 mm^3^, 4T1 cell-bearing mice were randomly divided into the following four groups (n = 5): 1) control group; 2) NIR laser-only group; 3) UCNPs@AgBiS_2_ group; and 4) UCNPs@AgBiS_2_ + NIR laser group. It was well known that the change in tumor volume and body weight within 14 days was the immediate performance of treatment effect and safety. During the treatment period, the body weight of each group maintained a steady increase (Fig. 5d), which indicated that the treatment of UCNPs@AgBiS_2_ with PDT/PTT had no obvious systemic toxicity. Taken together, the tumor growth curves of all groups are shown in Fig. 5e. Group 4 treated with UCNPs@AgBiS_2_ core-shell NPs under 808 nm laser irradiation caused the tumor volume to be sustainably inhibited. In contrast, the tumor volume of groups 1–3 displayed no tumor suppression during the observation period. To accurately observe the actual tumor size and weight, illustrative photographs of the final tumors were excised from mice after 2 weeks of treatment (Fig. 5f-g). The results showed that the tumors were significantly suppressed when treated with UCNPs@AgBiS_2_ NPs upon NIR laser exposure, which showed better therapeutic performance than the other three groups, demonstrating the efficient combined therapy of PDT/PTT. Additionally, the results of hematoxylin/eosin (H&E), TdT-mediated dUTP nick-end labeling (TUNEL) and 8-hydroxy-2-deoxyguanosine (8-OH-dG) staining of tumor slices showed that the group treated with UCNPs@AgBiS_2_ NPs upon NIR laser exposure had the highest level of tissue damage (Fig. 5h, and Fig. S18-19). In contrast, both the control, laser- and UCNPs@AgBiS_2_-treated groups showed low or no apparent apoptosis. To verify the effect of PDT, 8-OH-dG staining was used to prove the DNA damage caused by the generation of ROS, and the results showed that the treatment site had a large green fluorescence representing DNA damage. Fortunately, no major organs exhibited obvious tissue damage or unnatural inflammatory lesions, confirming that UCNPs@AgBiS_2_ core-shell NPs exhibited excellent biosafety (Fig. S20). Taken together, UCNPs@AgBiS_2_ NPs with high safety could be used for combined PTT-PDT of tumors.

## 4 Conclusions

In summary, UCNPs@AgBiS_2_ core-shell NPs were successfully fabricated by a facile chemical process. The photothermal conversion ability for AgBiS_2_-based NPs could be enhanced by the combination of UCNPs via the doping concentration of Nd ions, and the photothermal conversion efficiency of UCNPs@AgBiS_2_ core-shell NPs could be tuned from 14.7 to 45.0% due to efficient cross-relaxation pathways between Nd^3+^ ions and AgBiS_2_. Furthermore, The UCNPs endows strong upconversion emissions when the doped concentration of Nd ions is 1% in the inner core, which excites the AgBiS_2_ shell to produce ROS for PDT of cancer cells. Meanwhile, *in vitro* and *in vivo* experiments demonstrated that the UCNPs@AgBiS_2_ core-shell NPs obtained satisfactory therapeutic effects by combining PTT and PDT. Collectively, this work proved that UCNPs@AgBiS_2_ NPs were excellent photothermal agents and photosensitizers that could be used for high-efficiency PTT-PDT of cancer.

## Declaration of interest statement

We declare that we have no financial and personal relationships with other people or organizations that can inappropriately influence our work, there is no professional or other personal interest of any nature or kind in any product, service and/or company that could be construed as influencing the position presented in, or the review of, the manuscript.

## CRediT author contribution statement

**Zhaoyou Chu:** conceived and designed the experiments, performed experiments, discussed the results, wrote and revised the manuscript. **Tian Tian:** performed experiments. **Juan Yang:** performed experiments. **Benjin Chen:** performed experiments. **Hao Chen:** performed experiments. **Wanni Wang:** performed experiments, discussed the results, wrote and revised the manuscript. **Peiqun Yin:** discussed the results. **Xiaoping Xia:** conceived and designed the experiments, discussed the results, wrote and revised the manuscript. **Hua Wang:** conceived and designed the experiments, discussed the results, wrote and revised the manuscript. **Haisheng Qian:** conceived and designed the experiments, discussed the results, wrote and revised the manuscript, All authors discussed the results and commented on the manuscript.
