# Supplementary material for: Upconversion nanoparticles@AgBiS2 core-shell nanoparticles with cancer-cell-specific cytotoxicity for combined photothermal and photodynamic therapy of cancers
Source: Bioact Mater. 2022 Jan 10;17:71–80. doi: 10.1016/j.bioactmat.2022.01.010 (PMC8958283; doi:10.1016/j.bioactmat.2022.01.010)
Supplement: Multimedia component 1 [file mmc1.docx]

**Supporting Information**

**Upconversion nanoparticles@AgBiS_2_ core-shell nanoparticles with cancer-cell-specific cytotoxicity for combined photothermal and photodynamic therapy of cancers**

*Zhaoyou Chu^a^, Tian Tian^b^, Zhenchao Tao^c^, Juan Yang^a^, Benjin Chen^a^, Hao Chen^a^, Wanni Wang^a^, Peiqun Yin^a^, Xiaoping Xia^d,^*, Hua Wang ^b,^*, Haisheng Qian^a,^**

*^a^ School of Biomedical Engineering, School of Basic Medical Sciences, Anhui Provincial Institute of Translational Medicine, Anhui Medical University, Hefei, Anhui 230032, P. R. China*

*^b^ Department of Oncology, The First Affiliated Hospital of Anhui Medical University, Anhui Medical University, Hefei, Anhui 230032, P. R. China*

*^c^ Department of Radiation Oncology, The First Affiliated Hospital of USTC, Division of Life Sciences and Medicine, University of Science and Technology of China, Hefei, Anhui, 230031, China*

*^d^ Department of Obstetrics and Gynecology, Children’s Hospital of Anhui Medical University, Anhui Provincial Children’s Hospital, Hefei, Anhui 230051, P. R. China*

**Experimental Section**

**1. Materials**

Bismuth nitrate pentahydrate (Bi(NO_3_)_3_·5H_2_O), Thioacetamide (C_2_H_5_NS), Ethylene glycol (EG), 1-tetradecanol and dimethylsulfoxide (DMSO) were obtained from Sinopharm Chemical Reagent Co. Ltd. Silver nitrate (AgNO_3_, 99.8%) and 9, 10-Anthracenediyl-bis(methylene)-dimalonic acid (ABDA) were purchased from Aladdin Reagent (Shanghai, China). 3, 3′, 5, 5′-Tetramethylbenzidineand (TMB, 99.5%) were purchased from Shanghai xushuo Biotechnology Co., Ltd (Shanghai, China). 9-(diethylamino)benzo[a]phenoxazin-5(5H)-one (Nile Red), 3-[4,5-dimethylthiazol-2-yl]-2,5-diphenyltetrazolium bromide (MTT) was purchased from Macklin Biochemical Co. Ltd (Shanghai, China). Reactive Oxygen Species Assay Kit and Annexin V-FITC/PI Apoptosis Detection Kit were purchased from Beyotime (Shanghai, China). Dulbecco’s modified Eagle’s medium (DMEM), RMPI medium 1640, penicillin−streptomycin, fetal bovine serum (FBS), trypsin were purchased from Invitrogen, MEM Non-Essential Amino Acids (MEM NEAA) and Dulbecco’s phosphate-buffered saline (PBS) were purchased from Gibco (Grand Island, USA). All reagents were of analytical grade and used without further purification. Other chemicals are of analytic grade and used as received. The surface morphologies, phase, fluorescence, optical properties, X-ray diffraction (XRD) and X-ray photoelectron spectra (XPS) of these products were investigated carefully according to our previous protocol or instruments.

**2. Synthesis of UCNPs, UCNPs@AA-[Zn(OH)_4_]^2-^ and UCNPs@ZnS nanoparticles**

**The synthesis of UCNPs nanoparticles.** UCNPs (NaYF_4_:Yb_0.3_/Er_0.005_/Nd_x_ (X=0, 0.005, 0.01, 0.03, 0.05)@NaYF_4_:Nd_0.2_) were prepared through sequential growth method, take NaYF_4_:Yb_0.3_/Er_0.005_/Nd_0.01_@NaYF_4_:Nd_0.2_ as an example (Adv. Mater. Interfaces 2016, 3, 1500649). Typically, 0.685 mmol YCl_3_, 0.3 mmol YbCl_3_, 0.005 mmol ErCl_3_ and 0.01 mmol NdCl_3_ were added into the mixture of oleic acid (6 mL) and 1-octadecene (15 mL), heated to 100 ^o^C and kept for 30 min in vacuum with subsequent cooling to room temperature. A solution of 8.0 mmol NH_4_F (0.2964 g) and 5.0 mmol NaOH (0.2 g) in 10 ml methanol was added into the previous solution and heated to 100 ^o^C for 10 min, then to 300 ^o^C and kept it for 1 h under a nitrogen atmosphere. Subsequently, 3 mL Y_0.8_Nd_0.2_-oleate precursor was injected into the reaction system drop by drop and kept at 300 ^o^C for 1 h. Finally, the solution was cooled down to room temperature, the nanoparticles were precipitated with 10 ml ethanol and collected by centrifugation, and then re-dispersed in 6 mL cyclohexane. NaNdF_4_@NaYF_4_:Nd_0.2_ was similar to NaYF_4_:Yb_0.3_/Er_0.005_/Nd_0.01_@NaYF_4_:Nd_0.2_, except 1 mmol NdCl_3_ were added.

**The synthesis of UCNPs@AA-[Zn(OH)_4_]^2-^ core-shell nanoparticles.** Typically, 1 mmol Zn(NO_3_)_2_**·**6H_2_O and HMTA with an equal molar amount were added into 50 mL aqueous solution containing 0.3 mmol of CTAB and 0.2 mmol of AA under magnetic stirring to form clear solution. Subsequently, 1.8 mL of as-prepared hydrophilic UCNPs (ca. 0.63 mg) was added by dropwise into the previous clear solution and stirred for 5 min. The mixture was heated and kept at 85^o^C for 10 h. The final product (UCNPs@AA-[Zn(OH)_4_]^2-^ core-shell nanoparticles) was collected by centrifugation and washed with distilled water and absolute ethanol for several times.

**The synthesis of UCNPs@ZnS core-shell nanoparticles.** The UCNPs@ZnS core-shell nanoparticles were synthesized from UCNPs@AA-[Zn(OH)_4_]^2-^ core-shell nanoparticles by a sulfidation process, which was carried out in a simple self-made sulfidation apparatus using H_2_S gas. In a typical synthesis process, 0.04 g UCNPs@AA-[Zn(OH)_4_]^2-^ core-shell nanoparticles were heated to 50^o^C and H_2_S gas was introduced into the reaction vessel and kept at 50^o^C for 4 h.

**3. Photothermal performance of UCNPs@AgBiS_2_ nanoparticles**

To evaluate the photothermal performance, deionized water and UCNPs@AgBiS_2_ nanoparticles with different concentrations were irradiated for 3 min by a NIR laser (808 nm, 1 W cm^−2^). The dispersion temperature was monitored by an IRT camera (Ti400, Fluke, USA).

To calculate the photothermal conversion efficiency of UCNPs@AgBiS_2_ nanoparticles, 2 mL of UCNPs@AgBiS_2_ nanoparticles aqueous dispersion (100 µg mL^-1^) was continuously irradiated under the same condition until reaching a steady-state temperature. The laser was then shut off and the temperature decrease process was also recorded. The photothermal conversion efficiency (η) was calculated using equation (1) described by Roper**^1^**:

$\eta=\frac{hS\left( T_{max}-T_{surr} \right)-Q_{s}}{I(1-{10}^{{-A}_{808}})}$ (1)

where *h* is the heat transfer coefficient, *S* is the surface area of the container, *Tmax* is the maximum system temperature, *Tsurr* is the ambient surrounding temperature, *Qs* is the heat associated with the light absorbance of the solvent, *I* is the laser power (1 W cm^−2^) and *A808* is the absorbance of Sb2Se3 nanosheets at 808 nm. The value of *hS* is derived according to equation (2):

$\tau_{s}=\frac{m_{D}C_{D}}{hS}$ (2)

where $\tau_{s}$ is the sample system time constant, *mD* and *CD* are the mass (1 g) and heat capacity (4.2 J (g ºC) ^-1^) of deionized water, respectively. *Qs* is measured independently to be 12.6 mW using pure water (J. Phys. Chem. C 2017, 111, 3636-3641; Nano. Lett. 2011, 11, 2560-2566).

4. Hydroxyl radical (·OH) and singlet oxygen (^1^O_2_) detection

3,5,3,5-tetramethylbenzidine (TMB) was used to detect the generation of ROS. Typically, UCNPs@AgBiS_2_ was added to 2 mL of phosphate buffer (pH 4.0) containing TMB (0.4 mM), and irradiate with 808 nm NIR laser for 60 minutes, and the UV absorption of the solution at 650 nm was recorded using UV-Vis absorption spectrum.

**·**OH generation is studied by terephthalic acid (TAOH) which is more soluble in hot alkaline solutions. Typically, 50 ml of NaOH aqueous solution (0.2 M) was heated to boiling, and then 0.8307 g of TAOH was added with continuous stirring until the mixture formed a clear solution. After the addition of UCNPs@AgBiS_2_, the TAOH mixed solution was irradiated with 808 nm NIR laser for a certain period time (5, 10, 15, 20, 25 and 30 minutes), and finally the solution was measured using Edinburgh FLS980 fluorescence spectrometer at 425 nm.

^1^O_2_ generation is studied by 9,10-anthracenediyl-bis(methylene) dimalonic acid (ABDA). Typically, UCNPs@AgBiS_2_ was added to 2 mL of phosphate buffer (PBS, 0.1 M, pH 7.4) containing ABDA (80 mg mL^-1^), and irradiated with 808 nm NIR laser for a certain period time (0, 10, 20, 30, 40 and 60 minutes), and the UV absorption of the solution at 380 nm was recorded using ultraviolet-visible absorption spectrum.

5. Cytotoxicity experiment

Human Umbilical Vein Endothelial Cells (HUVECs) were purchased from ATCC, and 4T1 cells were purchased from Shanghai Cell Research Institute. The cytotoxicity of UCNPs@AgBiS_2_ heterostructures was determined by standard MTT (3-(4,5-dimethyl-2-thiazolyl)-2,5-diphenyl-2-H-tetrazolium bromide) assay using HUVECs and 4T1 were evaluated as normal cell and tumor cell models. Typically, 4T1 cells were seeded in a 96-well plate at 1×10^4^ cells/well, and then incubated with different concentrations of UCNPs@AgBiS_2_ (0, 10, 20, 40, 80, and 160 μg mL^-1^) for 12 hours. Subsequently, laser irradiation for different times (0, 1, and 3 min), adding MTT solution and incubating for 4 hours to form formazan. Finally, added 100 μL dimethyl sulfoxide to dissolve and measure the absorbance at 570 nm with a microplate reader to determine the relative cell viability.

6. *In Vivo* Antitumor Therapeutic Efficacy

All institutional and national guidelines for the care and use of laboratory animals were followed. All animal experiments were approved by the Ethical Committee of Anhui Medical University (approved number: LLSC20210077). All animal experimental protocols were performed following the guidelines established by the Association of Laboratory Animal Sciences and the Center for Laboratory Animal Sciences at Anhui Medical University. All animal experimental protocols are investigated carefully according to our previous protocol or instruments. A human breast cancer xenograft tumor model of mice were established by injecting 4T1 cells (5×10^6^) into the mammary fat pads of female BALB/c nude mice. The mice were used for subsequent experiments until the tumor size reached approximately 100 mm^3^, and the tumor volume was calculated based on the following equation: tumor volume (mm^3^) = 0.5 × (tumor length) × (tumor width)^2^.

**7. Statistical analysis.**

Statistical analysis was performed using a t-test. The differences were considered to be statistically significant for a p-value (*P < 0.05, **P < 0.01, ***P < 0.001, ****P < 0.0001).

**Supporting Figures**


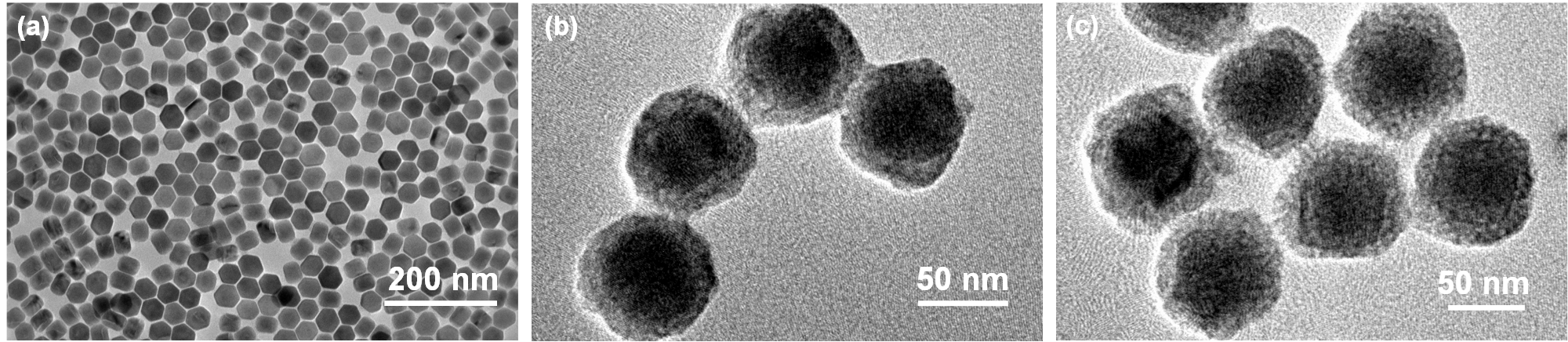


**Fig. S1.** (a-c) TEM images of NaYF_4_:Yb/Nd/Er@NaYF_4_:Nd upconversion nanoparticles, UCNPs@AA-[Zn(OH)_4_]^2-^ core-shell nanoparticles and UCNPs@ZnS core-shell nanoparticles, respectively.

**Fig. S2.** Elemental mapping images of Na, Er of UCNPs@AgBiS_2_ core-shell heterostructure.


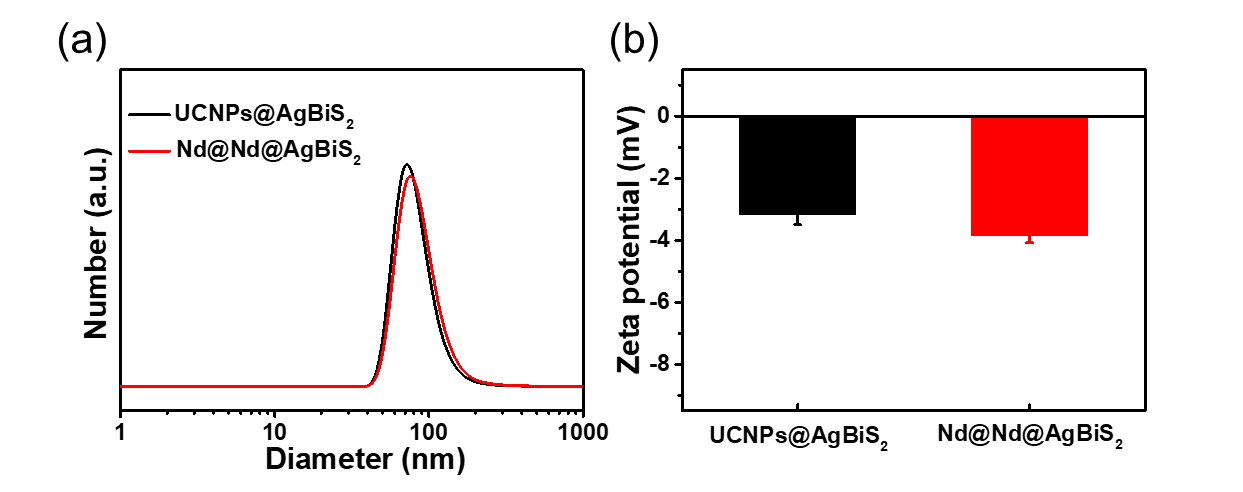


**Fig. S3.** (a, b) DLS and Zeta potential of various nanoparticles (UCNPs@AgBiS_2_ or Nd@Nd@AgBiS_2_).

**
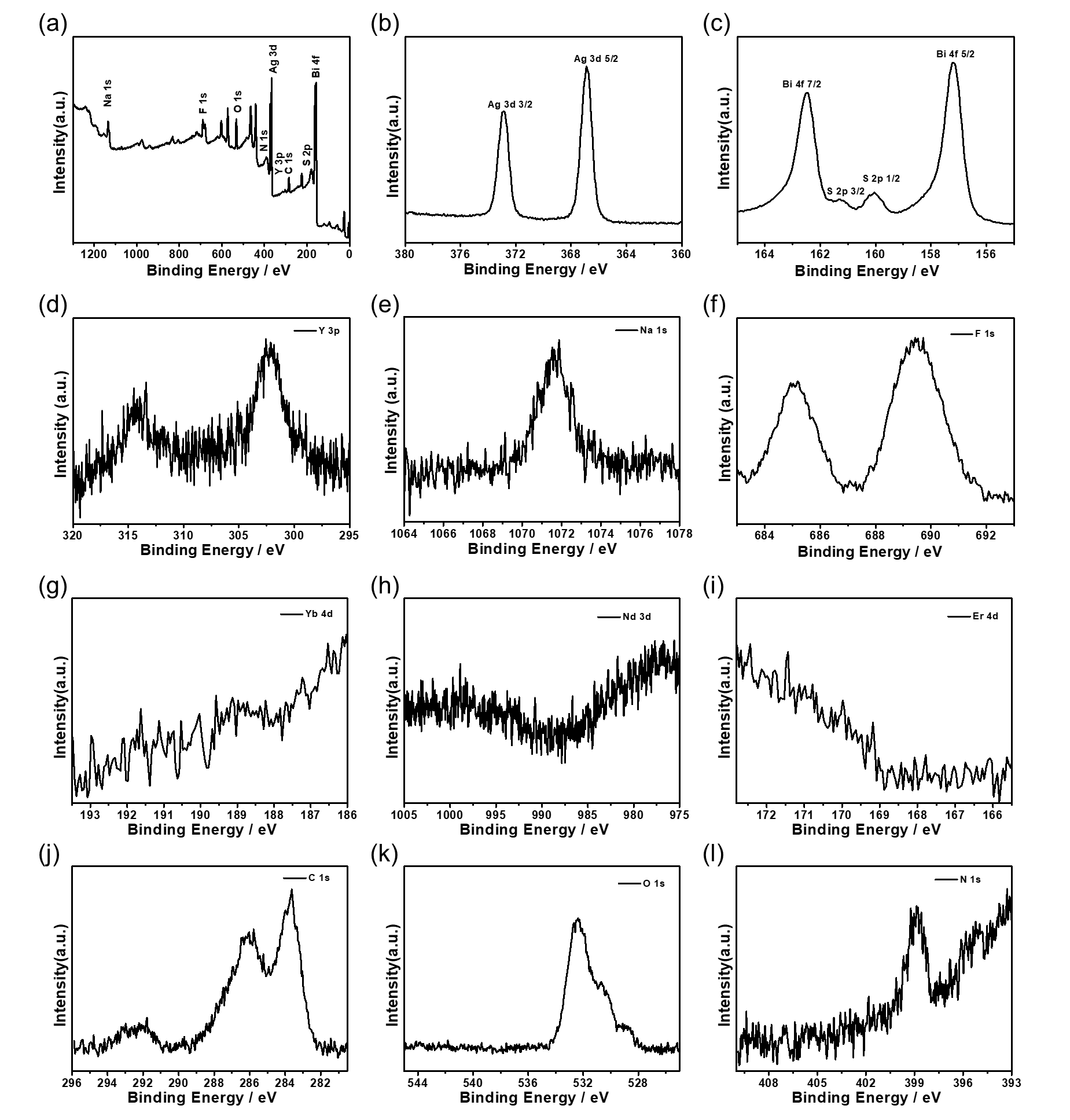
**

**Fig. S4.** X-ray photoelectron spectra of the product (UCNPs@AgBiS_2_): (a) a general survey; (b) Ag 3d; (c) Bi 4f and S 2p; (d) Y 3p; (e) Na 1s; (f) F 1s; (g) Yb 4d; (h) Nd 3d; (i) Er 4d; (j) C 1s; (k) O 1s; (l) N 1s; respectively.


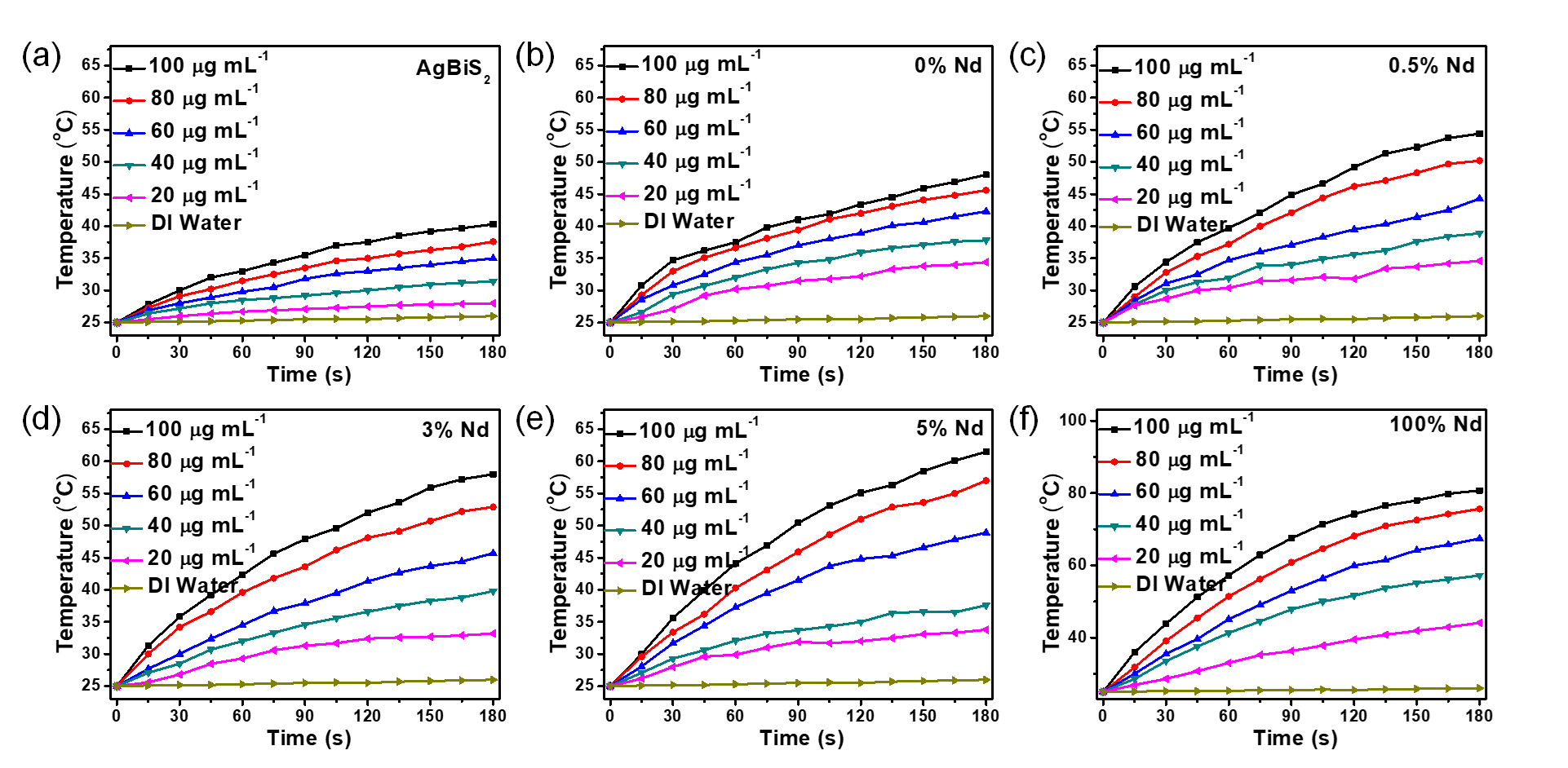


**Fig. S5.** The photothermal heating curve of different nanoparticles of various concentrations: (a) pure AgBiS_2_; (b) 0% Nd; (c) 0.5% Nd; (d) 3% Nd; (e) 5% Nd; (f) 100% Nd.


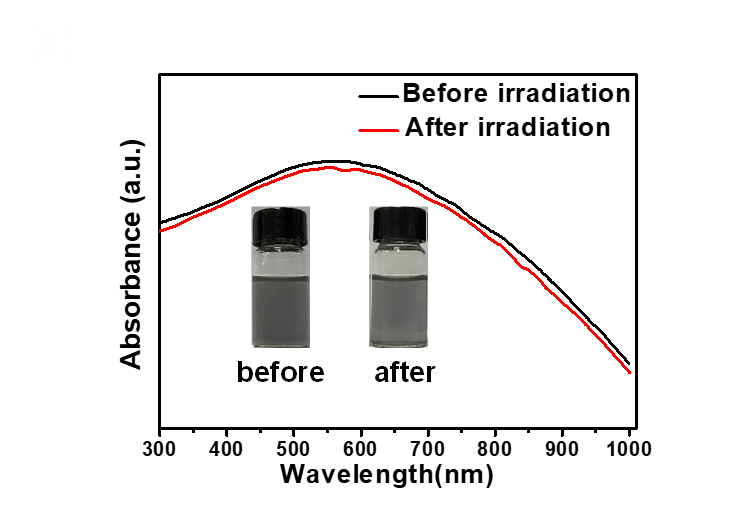


**Fig. S6.** UV-Vis-NIR absorption spectra of UCNPs@AgBiS_2_ dispersions before and after irradiation, the inset is the corresponding digital images of UCNPs@AgBiS_2_ nanocomposites before and after laser irradiation.


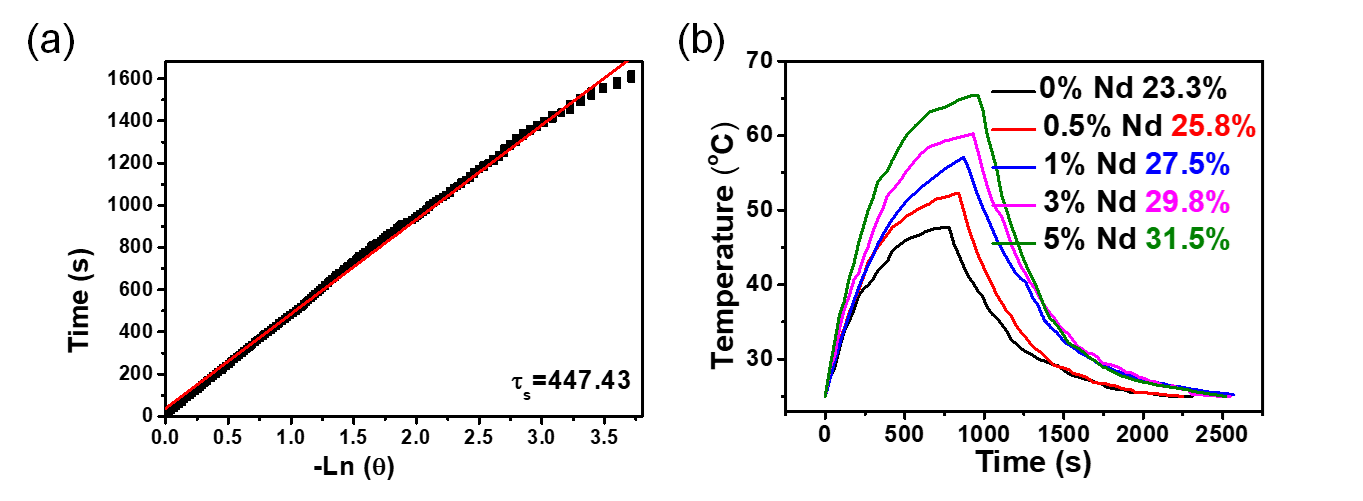


**Fig. S7.** (a) The fitting linear curve of time data vs -lnθ from the cooling period of UCNPs@AgBiS_2_ (1% Nd). (b) The heating/cooling curves of different nanoparticles with different Nd doping concentration under laser on/off.


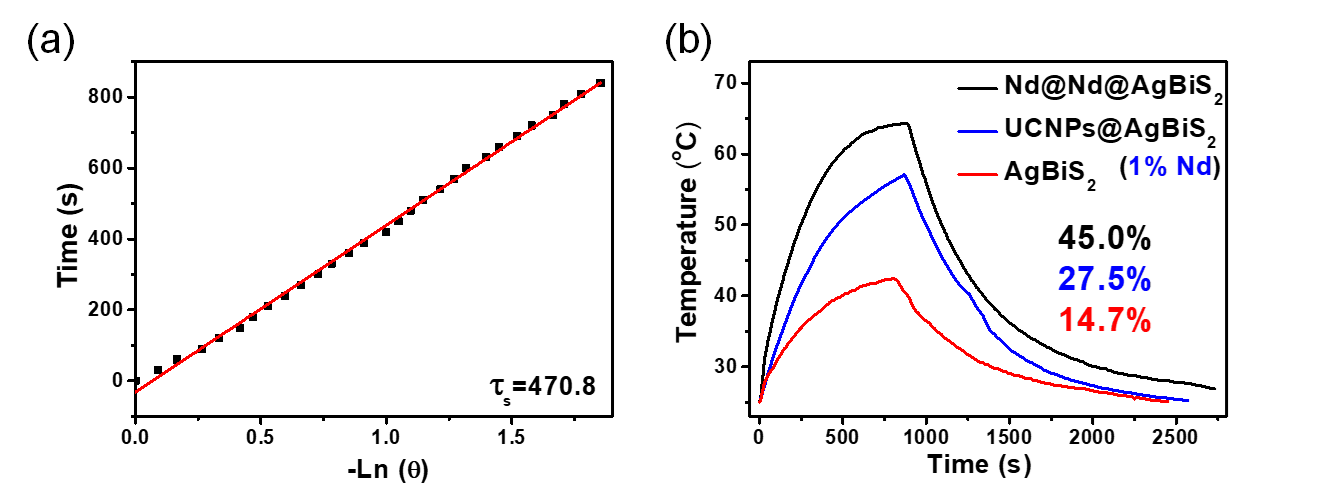


**Fig. S8.** (a) The fitting linear curve of time data vs -lnθ from the cooling period of Nd@Nd@AgBiS_2_ (100% Nd). (b) The heating/cooling curves of pure AgBiS_2_ nanoparticles, UCNPs@AgBiS_2_ (1% Nd) and Nd@Nd@AgBiS_2_ (100% Nd) under laser on/off.


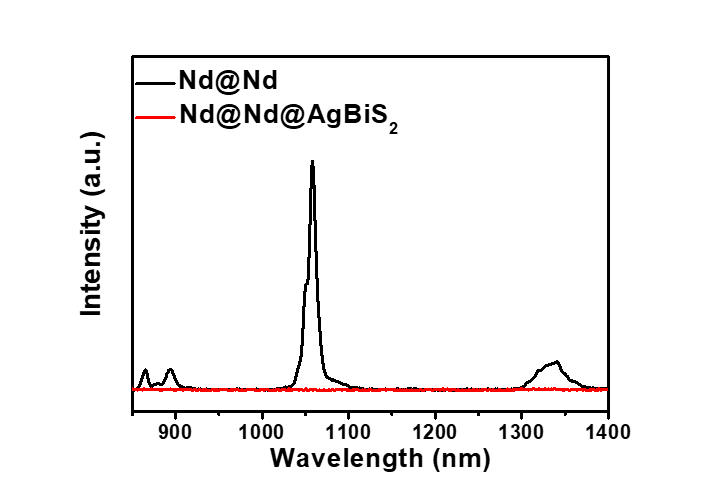


**Fig. S9.** The fluorescence spectra of the Nd@Nd nanoparticles and Nd@Nd@AgBiS_2_ core-shell nanoparticles, respectively.


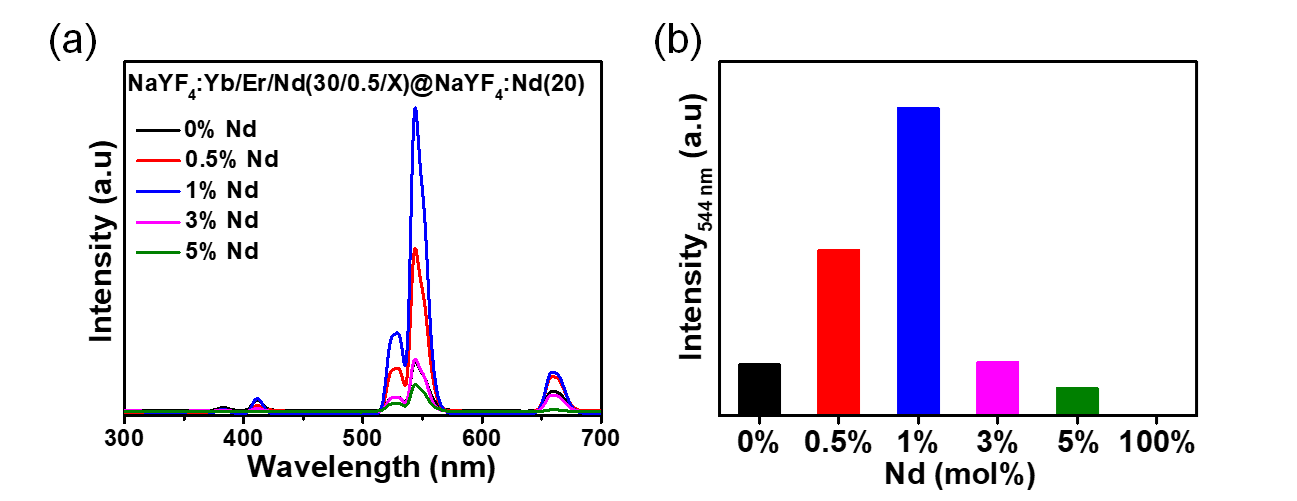


**Fig. S10.** (a, b) The fluorescence spectra and the intensity change of different nanoparticles with different Nd doping concentration.


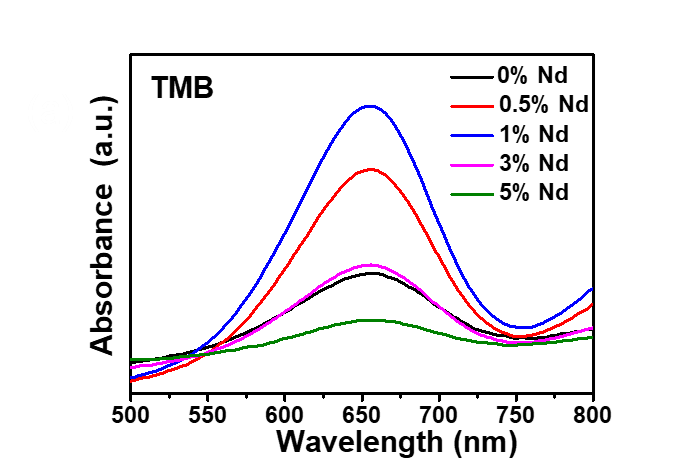


**Fig. S11.** The UV-Vis absorption spectra of TMB in the presence of UCNPs@AgBiS_2_ with different Nd doping concentration upon the irradiation of 808 nm laser.


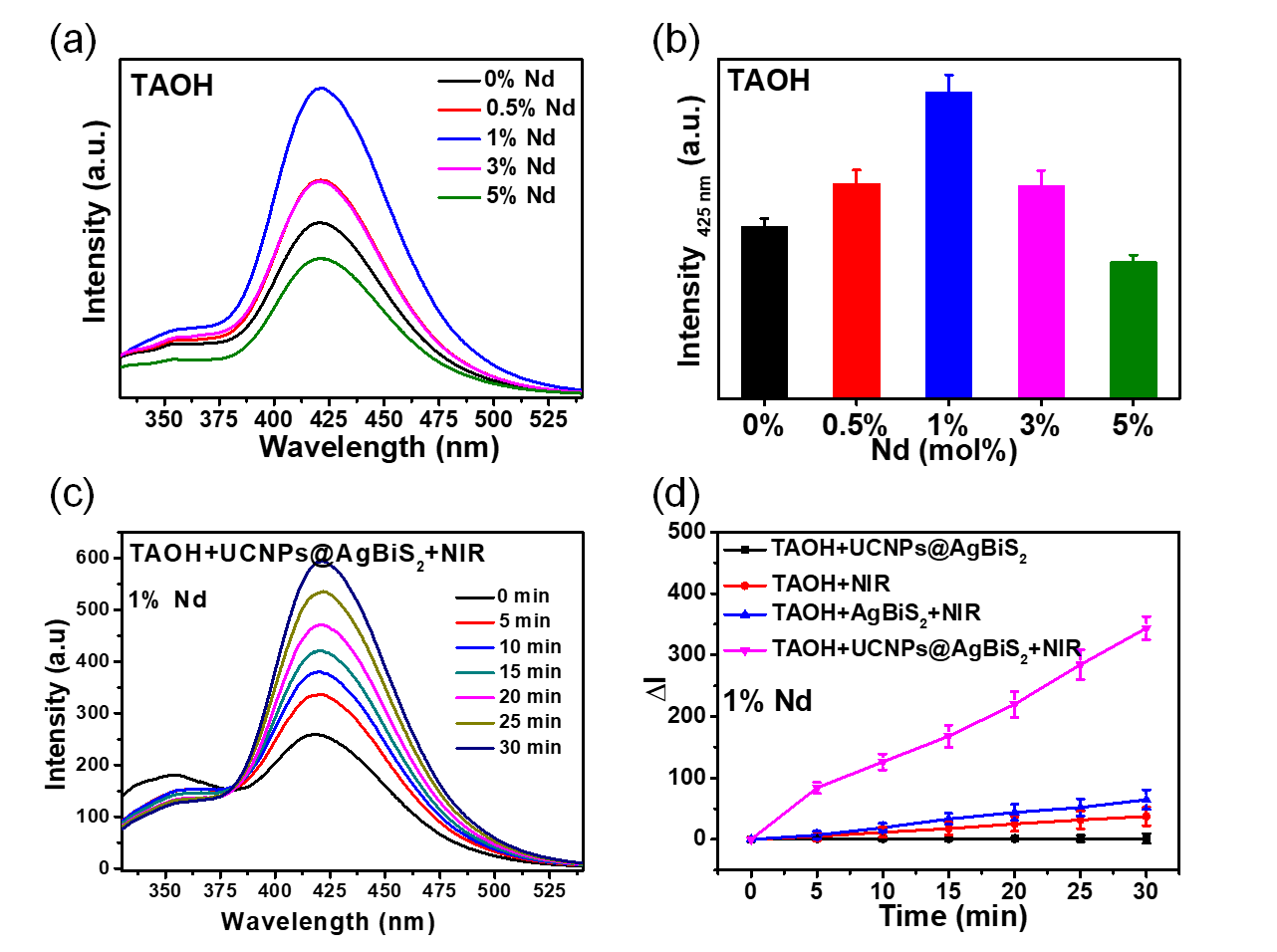


**Fig. S12.** (a, b) The fluorescence spectra and the intensity change of TAOH in the presence of UCNPs@AgBiS_2_ with different Nd doping concentration upon the irradiation of 808 nm laser, indicating the production of **·**OH species. (c, d) The fluorescence spectra and increase at 425 nm of TAOH solution in the presence of UCNPs@AgBiS_2_ upon the irradiation of 808 nm laser.


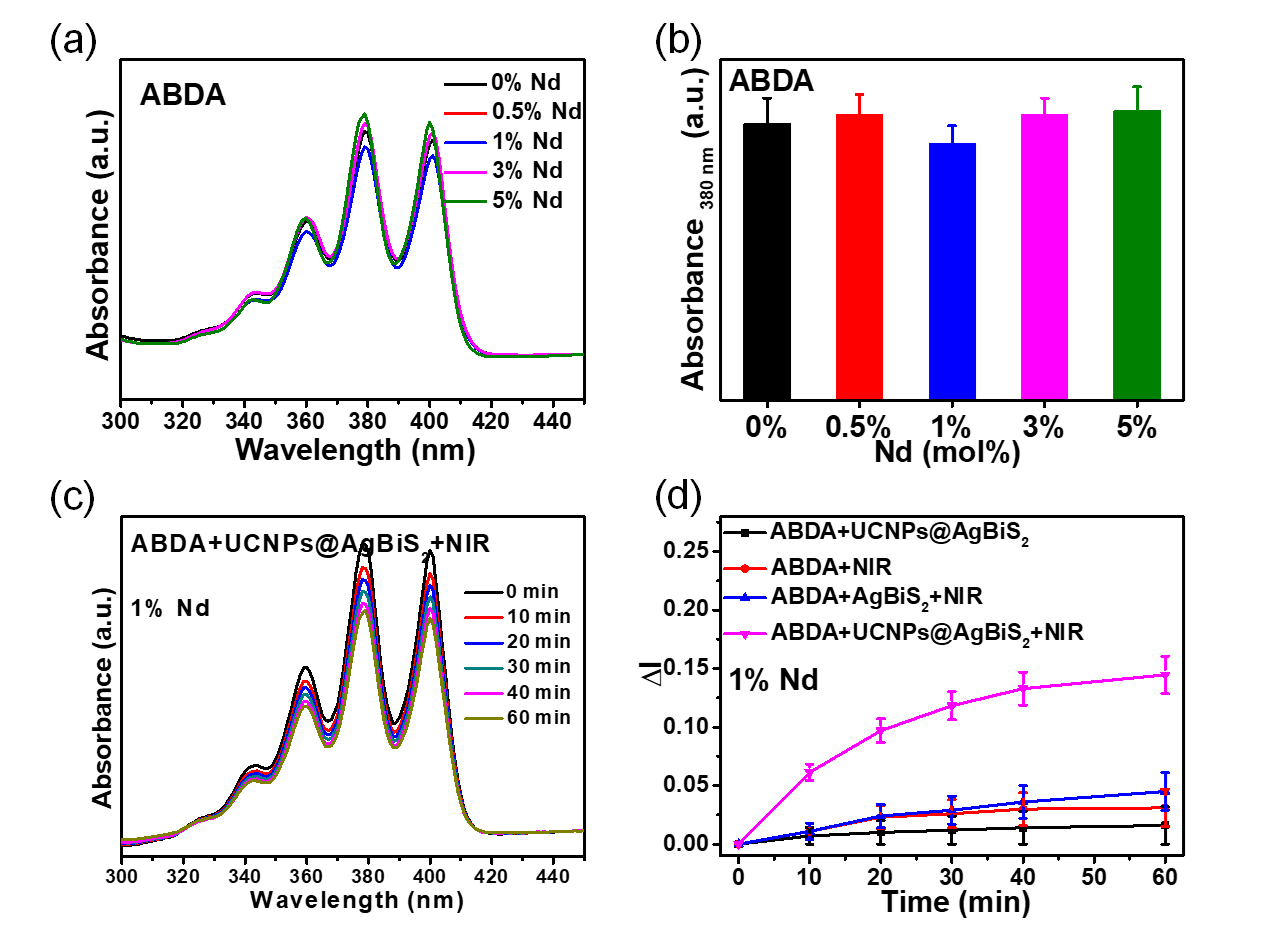


**Fig. S13.** (a, b) The UV-Vis absorption spectra and the absorbance change of ABDA in the presence of UCNPs@AgBiS_2_ with different Nd doping concentration upon the irradiation of 808 nm laser, indicating the production of ^1^O_2_ species. (c, d) The UV-Vis absorption spectra and decrease at 380 nm of ABDA solution in the presence of UCNPs@AgBiS_2_ upon the irradiation of 808 nm laser.


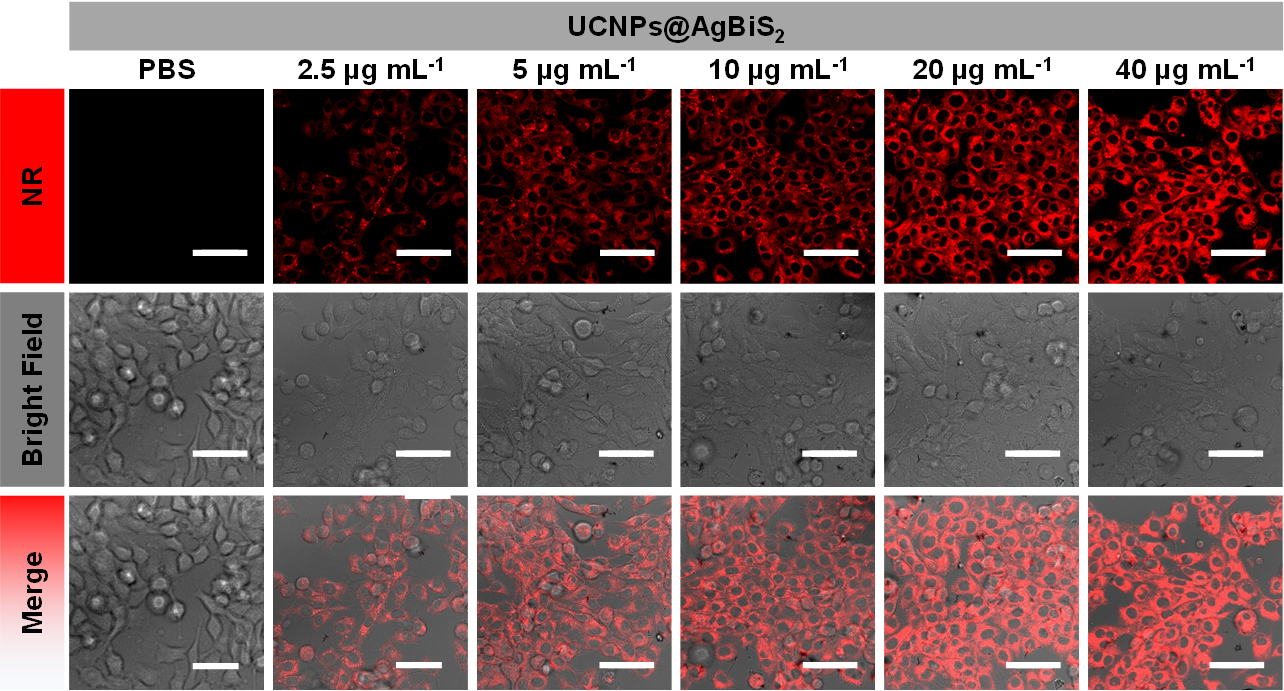


**Fig. S14.** CLSM images of 4T1 cells after 4 h incubation with UNP at different concentration (0, 2.5, 5, 10, 20, 40 μg mL^-1^). Scale bar = 50 µm.


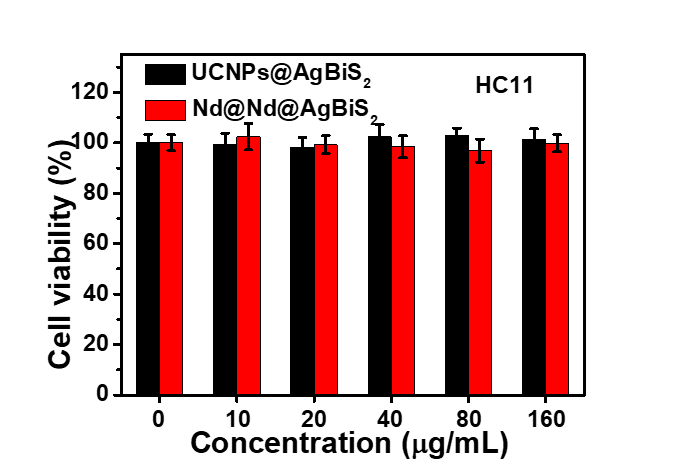


**Fig. S15.** Cell toxicity of HC11 after incubation with various concentrations of UCNPs@AgBiS_2_ and Nd@Nd@AgBiS_2_.


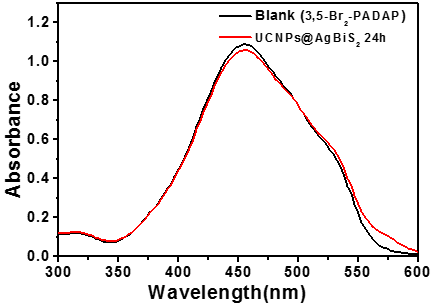


**Fig. S16.** Spectrophotometric method with 3,5-Br_2_-PADAP.


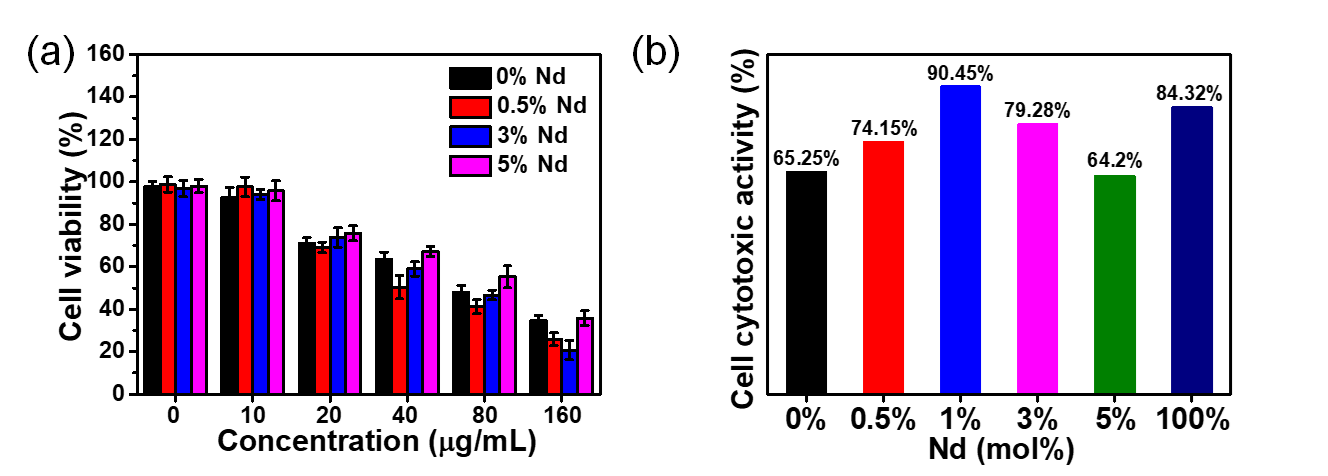


**Fig. S17.** (a-b) Apoptosis ablation of 4T1 cells incubated with various concentrations of different nanoparticles with different Nd doping concentration under irradiation (808 nm, 1.0 W cm^-2^).


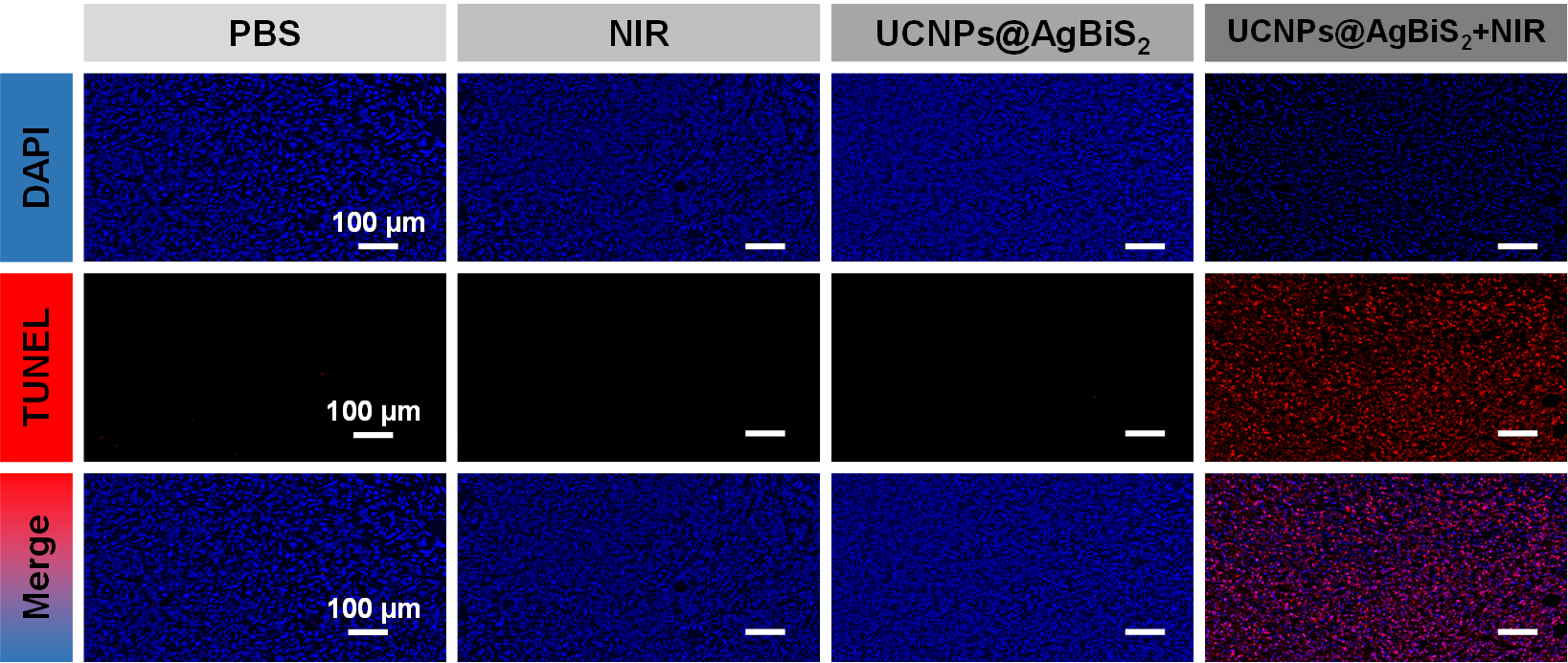


**Fig. S18.** TUNEL staining images of excised tumors of different treatments group on the 14th day.


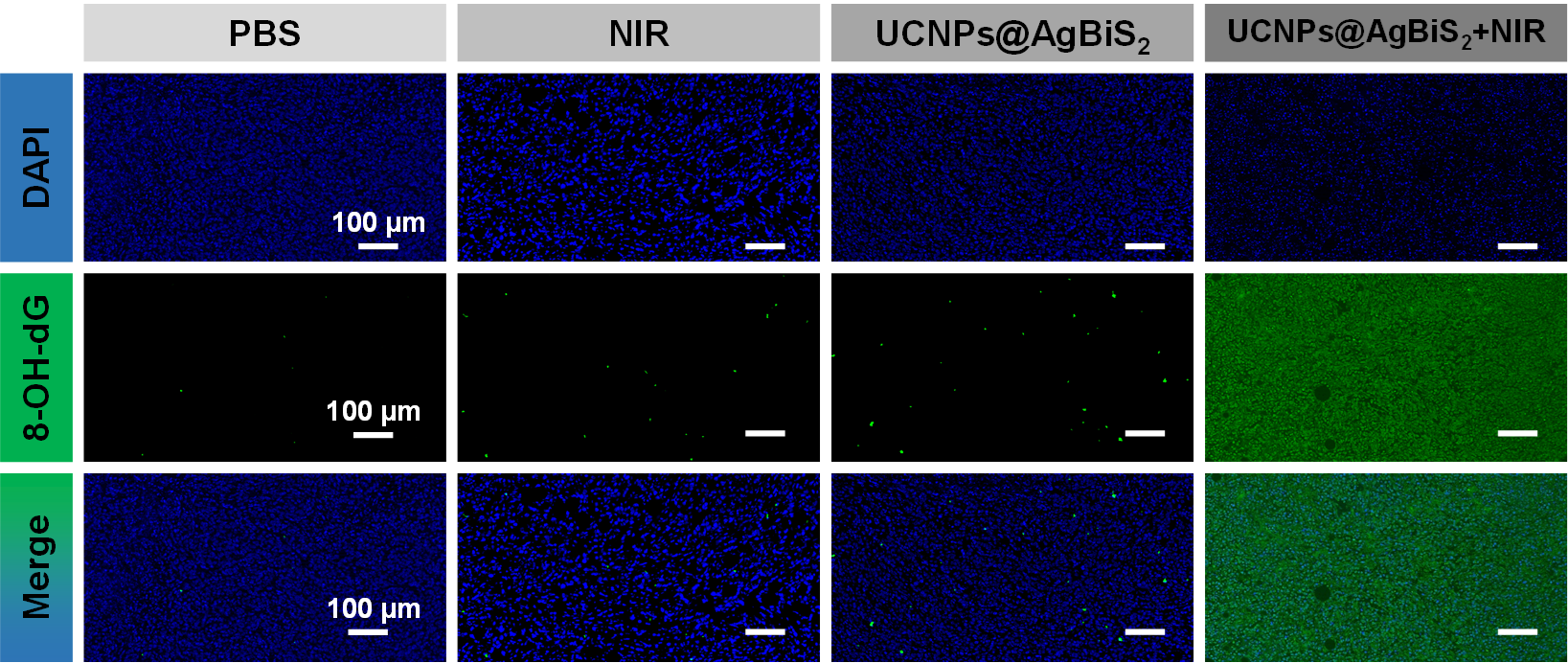


**Fig. S19.** 8-OH-dG staining images of excised tumors of different treatments group on the 14th day.


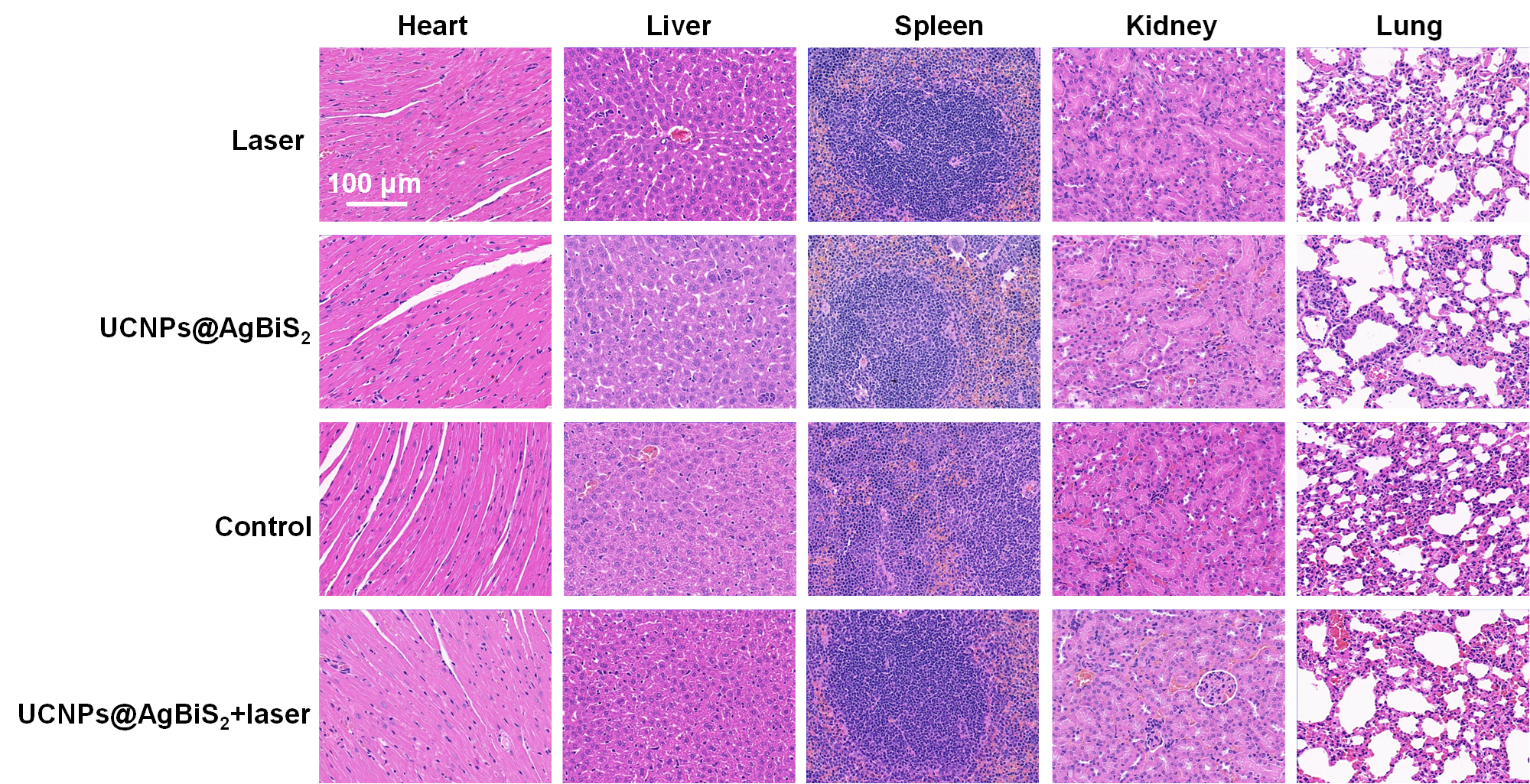


**Fig. S20.** Hematoxylin and eosin (H&E) stained images of major organs (heart, liver, spleen, kidney, and lung) of mice post-injection of UCNPs@AgBiS_2_ nanoparticles.

**Table S1.** Chemical composition of the as-prepared UCNPs@AgBiS_2_ composite nanoparticles from the elemental mapping analyses.

| Element | | Atomic % | |
| --- | --- | --- | --- |
| F K | | 16.15 | |
| Na K | | 1.28 | |
| S K | | 33.48 | |
| Y K | | 5.23 | |
| Ag K | | 19.52 | |
| Nd L | | 0.34 | |
| Er L | | 0.00 | |
| Yb L | | 1.38 | |
| Bi L | | 22.61 | |
| Totals | 100 | |  |

**Table S2.** Comparison of the photothermal conversion efficiency for different PTT agents.

| PTT agents | Photothermal conversion efficiency% | References |
| --- | --- | --- |
| Bi_2_S_3_ | 26.8 | 26 |
| Bi_2_S_3_ | 28.1 | 36 |
| UCNPs-Bi_2_Se_3_ | 29.97 | 27 |
| AgBiS_2_-TPP | 23.5 | 29 |
| AgBiS_2_-PEI | 21.3 | 29 |
| AgBiS_2_-PEI | 35.2 | 28 |
| AgBiS_2_ | 36.51 | 37 |
| UCNPs@AgBiS_2_ | 45 | This work |
